# Engagement of the TCR against an oncolytic virus generates a population of effector CAR T cells with potent antitumor activity

**DOI:** 10.1126/sciadv.aef5331

**Published:** 2026-06-05

**Authors:** Olivia Liseth, Elizabeth Appleton, Benjamin Kendall, Jill Thompson, Thanich Sangsuwannukul, Jason Tonne, Rosa Maria Diaz, Laura Evgin, Anton Patrikeev, Nicolas Sarbia, Shane Foo, Kevin Harrington, Masahiro Ono, Alan Melcher, Richard Vile

**Affiliations:** ^1^ Graduate School of Biomedical Sciences, Mayo Clinic, Rochester, MN, USA.; ^2^ Medical Scientist Training Program, Mayo Clinic, Rochester, MN, USA.; ^3^ Institute of Cancer Research, London, UK.; ^4^ Imperial College London, London, UK.; ^5^ Departments of Molecular Medicine and Immunology, Mayo Clinic, Rochester, MN, USA.; ^6^ Medical Genetics Department, University of British Columbia, Vancouver, BC, Canada.; ^7^ Basic and Translational Research Department, BC Cancer Research Institute, Vancouver, BC, Canada.; ^8^ Joan Reece Chair of Immuno-oncology, Comprehensive Cancer Centre, School of Cancer and Pharmaceutical Sciences, and School of Immunology and Microbial Sciences, Kings College London, London, UK.

## Abstract

Chimeric antigen receptor (CAR) T cell therapy faces many challenges against solid tumors including T cell exhaustion and poor CAR durability. Here, we show that engaging the CAR T cell endogenous T cell receptor (TCR) using an oncolytic virus enhances CAR T cell functionality, durability, and therapy. Upon combination therapy of solid tumors with CAR T cells and vesicular stomatitis virus (VSV), a subpopulation of antiviral, TCR-primed CAR T cells was generated with enhanced effector functions, altered activation states, and differential gene and protein expression when compared to non–TCR-primed CAR T cells. Single-cell RNA sequencing showed clonal expansion of anti-VSV CAR T cells and enhancement of effector-associated genes with VSV-mediated CAR T cell expansion. CD4 T cells played a pivotal role in the development of these TCR-primed CAR T cells. These results provide a strong rationale both for a novel use of systemic oncolytic virotherapy and for directly exploiting the CAR T cell TCR to fine tune the CAR T cell phenotype and function.

## Teaser

Clonal expansion through a high-affinity TCR alters downstream signaling and differentiation to promote CAR T cell cytotoxicity.

## INTRODUCTION

Chimeric antigen receptor (CAR) T cell therapy has revolutionized the care of patients with hematologic cancers. Seven CAR T cell therapies are currently US Food and Drug Administration approved for the treatment of plasma cell and lymphoid cancers (*1*). However, against solid tumors, CAR T cells face much more limited success for a variety of reasons including variable patient responses, antigen escape, tumor heterogeneity, limited CAR T persistence, and the uniquely immunosuppressive solid tumor microenvironment (*2*–*4*). Traditional human CAR T cell manufacturing entails extracting peripheral blood mononuclear cells from patients and engineering these peripheral blood mononuclear cells or a T cell–enriched pool to express a CAR through transduction with a lentivirus or retrovirus (*3*). This process induces expression of the CAR while retaining the endogenous T cell receptor (TCR) and its function. The CAR itself is designed to recognize tumor antigens in a major histocompatibility complex–independent fashion, transmitting activating signals via fusion of a single-chain variable fragment (scFv) to intracellular signaling domains borrowed from TCR signaling (*2*). In principle, the CAR acts like a second TCR, triggering T cell activation, proliferation, and effector functions in similar ways; however, many studies show that T cell activation through a CAR has a very different character than through a TCR (*5*–*7*). Therefore, depending on the source of the activating signal, CAR versus TCR, the effects on T cell phenotype and function are likely to be different.

Because of the potential role of the TCR in influencing CAR T cell phenotype, TCR engagement has been interrogated as a method to augment CAR T cell function. Early approaches attempted to use alloreactive T cells to engage the endogenous TCR and maintain in vivo CAR T cell survival. In mice, these alloreactive CAR T cells expanded upon infusion and exerted improved solid tumor control (*8*). In humans, preexisting antiviral T cells have been identified as a target population from which to make CAR T cells using anti–Epstein-Barr virus, cytomegalovirus, or Varicella Zoster virus T cells for CAR T cell manufacture. Although activation of the TCR was consistently proven to expand CAR T cell pools, the function and efficacy of the CAR therapies varied (*9*–*11*). It has been shown that while strong TCR interactions can potentiate the CAR function, weaker TCR antigens serve as antagonists, thereby suggesting a potential mechanism by which the TCR can be used to avoid anti-self CAR activity (*12*). Conversely, there is also a strong rationale to remove the endogenous TCR altogether, in an effort to generate an “off-the-shelf” CAR T cell product. These TCR knockout CAR T cells maintain cytotoxic activity in vivo while avoiding graft-versus-host disease (*13*, *14*). However, knocking out the native TCR was also correlated with diminished CAR T cell persistence (*15*). Given these conflicting approaches, we investigated whether it would be possible to exploit the native TCR of CAR T cells as a primary mediator of T cell expansion and activity and as a potential agonist for CAR T cell function.

In this study, we used oncolytic viruses (OVs) to generate immunodominant epitopes with which to engage and prime the endogenous TCR of CAR T cells. OVs selectively replicate in and kill tumor cells and create an inflammatory, immunogenic tumor microenvironment that is more conducive for T cell infiltration than the native tumor microenvironment (*16*–*18*). Consistent with our previous observations (*19*), we show here that combining CAR T cell therapy with the OV vesicular stomatitis virus (VSV) against solid tumors generates a population of CAR T cells that become primed against immunodominant epitopes of VSV, gain effector functions, and mediate long-term tumor cure when systemically boosted with a second dose of VSV. Here, we sought to understand the mechanisms through which this antiviral T cell priming alters CAR T cell function and expands populations of CAR T cells with enhanced cytotoxic capacities. We demonstrate that, instead of selecting for a population of preexisting antiviral T cells, a novel population of TCR-primed CAR T cells can be generated using a virus against which most people are not immune. Furthermore, this population of TCR-primed CAR T cells acquired a unique signaling profile, being paradoxically less activated while, at the same time, expressing increased levels of effector-associated proteins and transcripts and undergoing viral-mediated clonal expansion, to drive unique phenotypic features. These findings make a case for keeping, and using, the endogenous TCR in CAR T cells against strong, immunodominant, and boost-able epitopes.

## RESULTS

### Boosting TCR-primed CAR T cells promotes long-term survival and CAR T cell durability

**Fig. 1.**
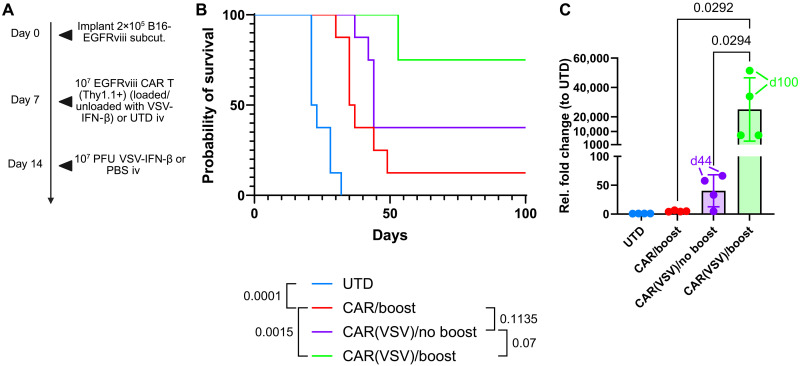
Coadministration of CAR T cells with VSV, followed by a VSV boost, mediates solid tumor cure and long-term CAR T cell durability in vivo. (**A** and **B**) Mice bearing subcutaneous B16EGFRviii tumors were treated with either 10^7^ EGFRviii CAR T cells loaded with VSV-IFN-β (MOI 1) at 4°C for 1 hour [CAR(VSV)], 10^7^ EGFRviii CAR T cells, or 10^7^ UTDs on day 7 post–tumor implantation and then boosted with either PBS or 10^7^ PFU VSV-IFN-β on day 14. The survival of mice was recorded (*n* = 8). A log-rank Mantel-Cox test of statistical significance was performed with Bonferroni correction for multiple comparisons. iv, intravenously. (**C**) Mouse splenocytes were collected at the end point (tumor at a 1-cm diameter in any dimension), splenocyte RNA was isolated, and qRT-PCR was run to determine the prevalence of systemic CAR T cells remaining after therapy. Statistical significance was calculated using a one-way ANOVA with Tukey’s for multiple comparisons. d, day.

To demonstrate the therapeutic benefit of dual CAR/VSV therapy, we challenged C57BL/6 mice with subcutaneous B16F10 melanoma tumors lentivirally transduced to express the EGFRviii protein (B16EGFRviii), followed by a prime-boost treatment scheme using EGFRviii CAR T cells and VSV expressing murine interferon-β (VSV-IFN-β) (Fig. 1A and fig. S1). For the prime-boost treatment, CAR T cells were loaded with VSV through ex vivo coincubation before intravenous injection and then boosted in vivo with VSV intravenously to reengage VSV-specific T cells. VSV expressing murine IFN-β was used for improved tumor-selective infection with intravenous administration, as IFN-β limits viral infection of normal tissues. Whereas treatment of B16EGFRviii tumors with non–CAR-expressing T cells [untransduced T cells (UTDs)] resulted in all mice succumbing to tumor growth by 32 days postimplantation, treatment with nonloaded CAR T cells and a viral boost, or with VSV-loaded CAR T cells without a boost, significantly improved survival. We have also confirmed previously no survival benefit of intravenous VSV-IFN-β alone (*20*). Furthermore, when VSV-loaded CAR T cells were administered in combination with a follow-up VSV boost, six of eight mice survived beyond 100 days (Fig. 1B and fig. S2). RNA isolated from mouse spleens for quantitative reverse transcription polymerase chain reaction (qRT-PCR) detection of circulating CAR T cells at the point of euthanasia, using the UTD-treated group as a reference, showed that VSV loading of CAR T cells, and a VSV boost, promoted significantly improved CAR T cell in vivo durability than either loading or a boost alone (Fig. 1C). Of the mice monitored, the two that survived >100 days tumor-free also showed the highest fold change of CAR vector persistence relative to the UTD-treated group (green arrows, survival to day 100; Fig. 1C). Similarly, the two mice that survived the longest in the group cotreated with VSV but did not receive a boost also showed the highest level of CAR T cell durability (purple arrows, survival to day 44; Fig. 1C). Together, these data suggest that in vivo CAR T durability induced by TCR engagement correlated with tumor rejection and survival.

### VSV-CAR T cotreatment promotes CD8 tumor infiltration and skews CAR T cells toward an effector phenotype

**Fig. 2.**
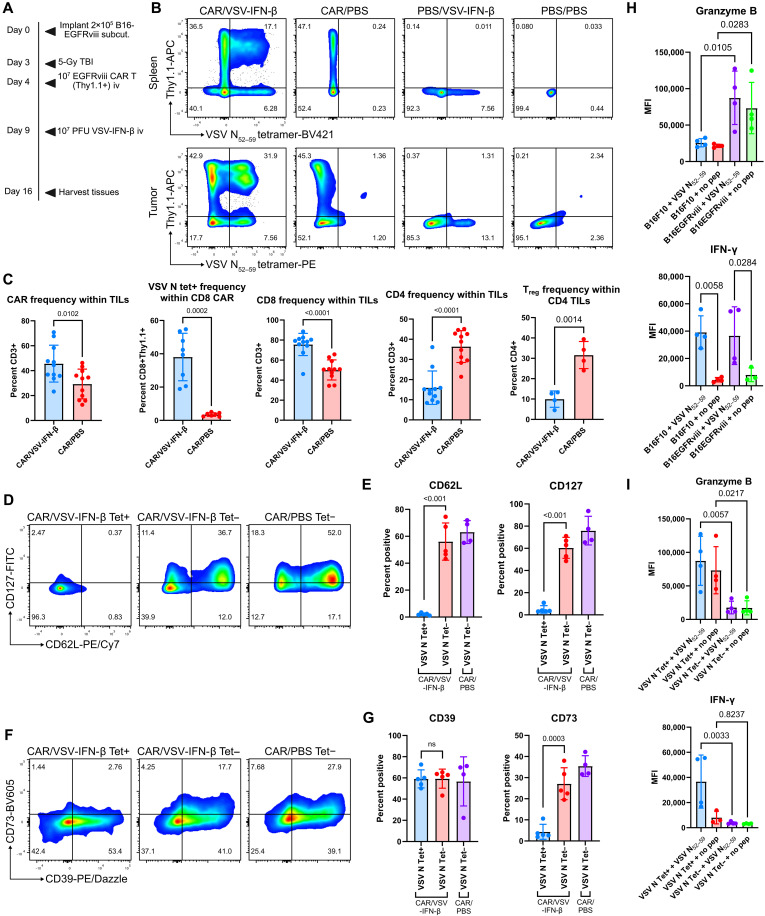
Cotreatment with CAR T cells and VSV for solid tumors promotes CAR T cell and overall CD8 tumor infiltration while generating a subpopulation of phenotypically and functionally unique TCR-primed CAR T cells. (**A**) C57BL/6 mice (Thy1.2+) bearing subcutaneous B16EGFRviii tumors were lymphodepleted on day 3 with 5-Gy total body radiation, treated on day 4 with 10^7^ EGFRviii CAR T cells, and treated on day 9 with PBS or VSV-mIFN-β intravenously (10^7^ PFU). Spleens and tumors were harvested on day 16. (**B**) Representative flow plots of CAR (Thy1.1+) and VSV N–reactive (tetramer+) populations in the spleens and tumors of mice. (**C**) Frequency of T cell populations within TILs. Data are pooled from two or three independent experiments (*n* = 4 to 11). (**D** to **G**) Representative flow plots and quantification illustrating CD127, CD62L, CD39, and CD73 expression within the spleen tetramer+ CAR T population (*n* = 4 or 5). ns, not significant. (**H**) CAR/VSV splenocytes were cultured for 16 hours with B16F10 or B16EGFRviii at a 10:1 ratio (effector:target) with or without VSV N_52–59_ peptide. In the final 4 hours of culture, BD GolgiPlug was added. The median fluorescence intensity (MFI) of granzyme B and IFN-γ using intracellular flow antibodies was quantified. (**I**) Under the same conditions as (H), granzyme B and IFN-γ production was measured in TCR-primed versus non–TCR-primed CAR cocultured with B16EGFRviii with or without VSV N_52–59_ peptide (*n* = 4). Statistical significance was calculated using a one-way ANOVA with Tukey’s for multiple comparisons or an unpaired two-tailed *t* test for two-sample comparisons.

Following either loading of CAR T cells with VSV-IFN-β (Fig. 1) or sequential therapy of intravenous CAR T cells and intravenous VSV-IFN-β (Fig. 2A and fig. S3), a subpopulation of CAR T cells expanded with specificity against an epitope of the immunodominant nucleocapsid protein of VSV, VSV N_52–59_ (RGYVYQGL, hereafter abbreviated to VSV N), comprising between about 20 and 50% of tumor-infiltrating CD8 CAR T cells (Fig. 2, B and C). A Thy1.1 reporter was used to detect CAR T cells in Thy1.2+ C57BL/6 mice. Combination of CAR T cells with VSV-IFN-β significantly increased overall CAR infiltration into tumors while skewing the T cell population toward CD8 and away from CD4 (Fig. 2C). Viral therapy also decreased the proportion of Foxp3+ T regulatory cells within the tumors as compared to CAR T cell therapy alone (Fig. 2C). The overall intratumoral T cell profile suggests that combining VSV with CAR T immunotherapy both promotes CAR T cell tumor infiltration and cultivates a more CD8-dominant, antitumor lymphocyte profile.

Using an H-2K^b^ VSV N_52–59_ fluorophore-conjugated tetramer (Tet) to detect CAR T cells with a TCR specific to the immunodominant VSV N epitope, we observed that CAR T cells that had been primed and expanded against VSV N (VSV N Tet+) acquired a significantly different phenotype both from VSV N Tet− CAR T cells within the same mice and from CAR T cells cotreated with vehicle control [phosphate-buffered saline (PBS)]. These TCR-primed CAR T cells expressed significantly lower levels of CD62L, CD127, and CD73, all markers of naïve- or memory-like CD8 T cells (Fig. 2, D to G), suggesting that antigen-specific TCR priming and signaling promoted the acquisition of an effector phenotype by CD8 CAR T cells compared to their non–TCR-primed counterparts. The observation that VSV N Tet− CD8 CAR T cells within the same viral microenvironment did not undergo the same degree of effector polarization highlights the importance of TCR signaling and differential T cell activation through both the CAR and TCR in altering the CAR T cell phenotype in response to a combination with an OV.

### TCR-primed CAR T cells demonstrate superior effector functions

Among total CD8 CAR T cells (both VSV N Tet+ and Tet−) from splenocytes of mice receiving CAR/VSV cotreatment, the presence of the CAR target antigen (EGFRviii) induced granzyme B production, while the TCR antigen (VSV N_52–59_) induced IFN-γ (Fig. 2H). When comparing TCR-primed (Tet+) to Tet− CD8 CAR T cells, the TCR-primed CAR generated higher levels of granzyme B and IFN-γ compared to their non–TCR-primed counterparts in response to both CAR target–expressing B16EGFRviii tumor cells and the TCR-activating VSV N_52–59_ peptide, with superior CAR granzyme B production even in the absence of the TCR antigen (Fig. 2I). These findings demonstrate that T cell activation through both a CAR and TCR generates distinct effector properties compared to CAR signaling alone.

### TCR priming in CAR T cells correlates with decreased T cell activation via Nr4a3

**Fig. 3.**
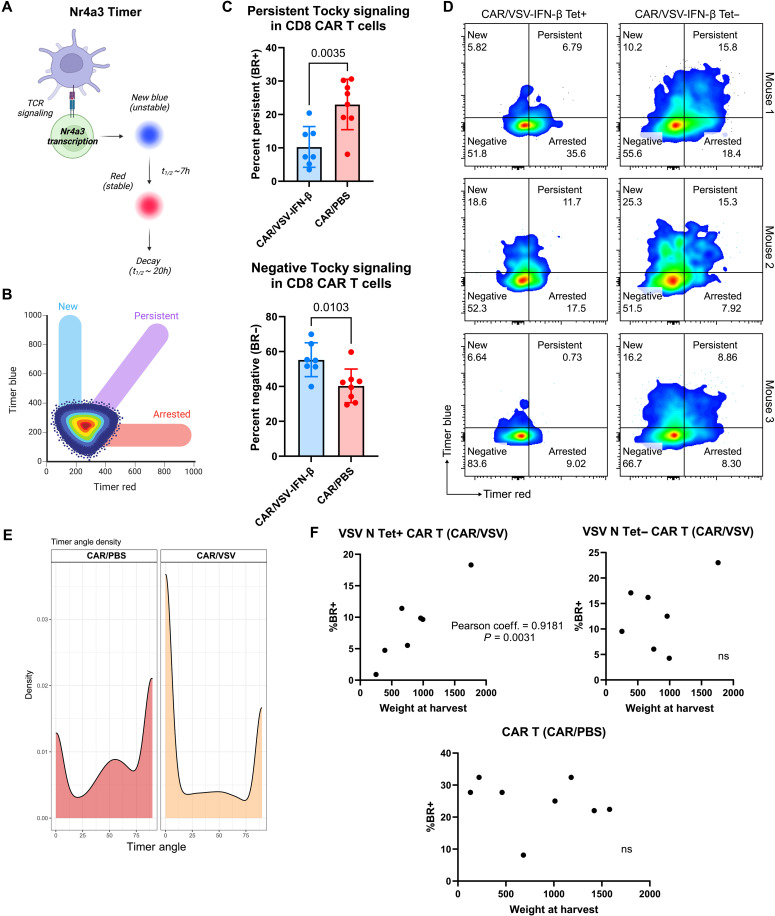
TCR engagement and priming of tumor-infiltrating CD8 CAR T cells alter their T cell activation profile toward a paradoxically less activated state. (**A**) Schematic depicting Timer protein expression downstream of Nr4a3 transcription and fluorescent protein half-lives. h, hours. (**B**) Flow plot characterizing the Tocky phenotype (new, arrested, and persistent) depending on the proportion of blue, red, or blue + red (purple). Adapted from Bending *et al.* (*21*) (CC BY 4.0; https://creativecommons.org/licenses/by/4.0/). (**C**) Intratumoral CD8 CAR T cells were analyzed using flow cytometry to determine levels of persistent or negative Tocky signaling between tetramer+ versus tetramer− cells. Data are pooled from two independent experiments (*n* = 6 to 8). (**D**) Representative flow plots of Timer protein expression between intratumoral TCR-primed (Tet+) versus non–TCR-primed (Tet−) CAR T cells from CAR/VSV–treated mice. (**E**) Density of cells populating each Timer angle (0, new; 90, arrested). (**F**) Plots correlating tumor weight at harvest in milligrams to the frequency of intratumoral blue-red+ CAR T cells (*n* = 7 or 8). Statistical significance was calculated using an unpaired two-tailed *t* test for two-sample comparisons or simple linear regression for correlative data.

The level of T cell activation was then interrogated using a reporter of Nr4a3, a protein downstream of NFAT (nuclear factor of activated T cells), one of the main pathways through which TCR signaling executes T cell function and activation. We used the novel Timer of cell kinetics and activity (Tocky) transgenic mouse model, which uses Timer protein as a reporter of Nr4a3 transcription (see Materials and Methods) (Fig. 3, A and B) (*21*). While the Tocky model was intended for use with TCR signaling, we confirmed that CAR binding also triggered Timer protein expression, leading to increased blue and red fluorescence upon Tocky EGFRviii CAR T cell coculture with B16EGFRviii targets (fig. S4). We then used Tocky splenocytes to generate EGFRviii CAR T cells for in vivo combination therapy with VSV-IFN-β, as outlined in Fig. 2A. Paradoxically, despite the addition of the highly inflammatory VSV, CD8 CAR T cells cotreated with VSV displayed decreased levels of Timer protein expression at 7 days post–VSV administration, reflecting a reduction in persistently engaged CAR T cells and an increase in CAR T cells with no Timer protein expression (Fig. 3C). When Timer protein profiles were subdivided into VSV Tet+ versus Tet− CD8 CAR T cells within the CAR/VSV group, once again, the Tet+ CAR T cells demonstrated a more negative, less persistent signature (Fig. 3D). Analysis of Timer protein angle (new, 0°; arrested, 90°) also revealed that a greater proportion of the CAR T cells cotreated with VSV had a new Tocky signature, characterized by a Timer angle closer to 0^0^ (Fig. 3E). Functionally, decreased persistent activation of the TCR-primed CAR T cells correlated with improved tumor control (decreased tumor size at day 16 harvest). These data suggest that the intensity of NFAT activation in CAR T cells is negatively correlated with T cell cytotoxicity and tumor control in our model. This relationship only held true for TCR-primed CAR and not the VSV N Tet− CAR or non–VSV-engaged CAR (cotreated with PBS) (Fig. 3F).

### CyTOF reveals distinct effector protein expression within CD8 TCR-primed CAR T cells

**Fig. 4.**
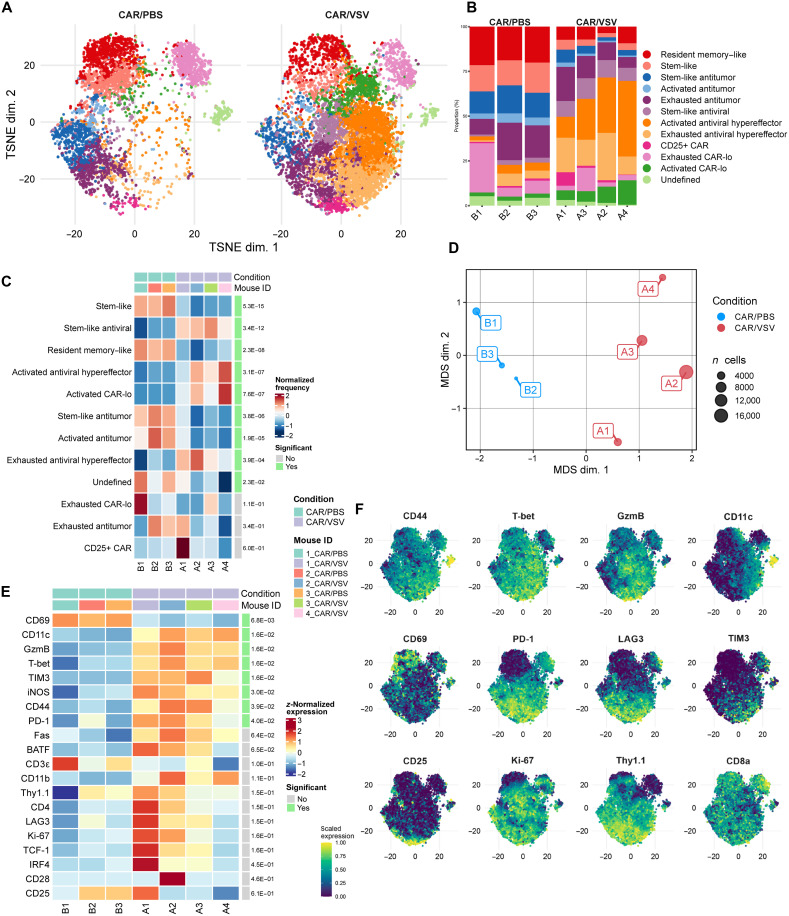
Population-wide differences in protein expression arise during CAR/VSV cotreatment to enrich for hypereffector CAR T cells. CyTOF was performed on tumor samples from day 16 of the experiment outlined in Fig. 2A after enriching for CD45+ cells using magnetic beads. (**A**) Overall TSNE plot for each treatment group combining four mice from the CAR/VSV samples and three mice from the CAR/PBS samples. (**B**) Relative population abundances for all clusters divided by sample. (**C**) Heatmap displaying the frequency of each sample within each cluster with *P* values displayed on the right-most column depicting statistically significant frequencies. (**D**) Multidimensional scaling analysis of CyTOF data from intratumoral CD8 CAR T cells demonstrating population differences between CAR/VSV– and CAR/PBS–treated mice. (**E**) Heatmap depicting the frequency of individual protein marker expression between samples, listed in order of *P* value. (**F**) TSNE plots combining all samples across treatment groups colored by expression of individual markers of interest.

Using the treatment scheme from Fig. 2A, t-distributed stochastic neighbor embedding (TSNE) plots generated using *k*-means clustering on CD8 CAR T cell–filtered data from cytometry by time of flight (CyTOF) on CD45-enriched samples from the tumors of mice treated with CAR/VSV or CAR/PBS demonstrated distinct differences between CD8 CAR T cells cotreated with PBS versus VSV-IFN-β (Fig. 4A), consistent across individual samples (fig. S5A). Cotreatment with VSV-IFN-β led to a significant increase in the abundance of antiviral T cells, including stem-like, activated, and exhausted cells, with a relative reduction in antitumor CD8 CAR (Fig. 4, B and C). Antiviral T cells were defined by their exclusive abundance in the CAR/VSV-IFN-β condition and increased CD11c and CD11b expression, integrins typically associated with dendritic cells but whose up-regulation on CD8 T cells is associated with viral reactivity (*22*). Antitumor T cells were those present in the CAR/PBS condition (without viral influence) and shown to be activated via increased CD44 expression, moderate granzyme B, and increased programmed cell death protein 1 (PD-1) levels. Stem-like clusters were defined as those expressing Ki-67 (proliferative potential) with intermediate CD44 as well as PD-1 and TCF-1 (T cell factor 1) positivity, with activated T cells higher in CD44 and granzyme B but without significant up-regulation of exhaustion markers LAG3 (lymphocyte-activation gene 3) and TIM3 (T cell immunoglobulin and mucin domain-containing protein 3) coexpressed with PD-1 used to define the exhausted phenotype (*23*, *24*). A resident memory–like cluster was identified, defined by high CD69 expression and relatively low Ki-67. A cluster of CD8 CAR was observed with particularly high CD25 expression, labeled CD25+ CAR, and two additional clusters expressed particularly low levels of Thy1.1, our CAR marker. These were subsequently labeled either activated or exhausted CAR-lo depending on levels of exhaustion markers within each cluster (fig. S5C).

Together, multidimensional scaling analysis revealed significant differences in overall protein profiles of CAR T cells cotreated with VSV versus PBS. Samples from CAR/PBS–treated mice (B1, B2, and B3) clustered together and were separate from those treated with CAR/VSV-IFN-β (A1, A2, A3, and A4) (Fig. 4D). Within the animals receiving virus, CD11c, granzyme B, T-bet, TIM3, iNOS (inducible nitric oxide synthase), CD44, and PD-1 were the most up-regulated markers, with only CD69 being significantly up-regulated in CAR/PBS mice (Fig. 4E and fig. S5B). This significant up-regulation of effector- and activation-associated proteins led us to designate our antiviral cluster as “hypereffector.” This was rationalized by the significant up-regulation of effector proteins in antiviral CAR clusters, not present in CAR alone (PBS). Although not significant, other markers associated with effector differentiation in CD8 T cells were also up-regulated with VSV cotreatment, including BATF (basic leucine zipper ATF-like transcription factor) and IRF4 (interferon regulatory factor 4), in addition to Ki-67 and TCF-1, which suggest greater proliferative potential. Individual TSNE plots for markers of interest emphasize the unique gradients of protein expression across clusters, with particularly increased T-bet, granzyme B, and CD11c throughout antiviral clusters (Fig. 4F).

### CD4 population changes promote an antiviral CD8 CAR T cell response

Antitumor activity can also be mediated by CD4 T cells via CD8 T cell priming and cytokine production (*25*, *26*). Using our CyTOF data gated on live CD45+CD3+CD4+ T cells, we found that the overall CD4 populations within the tumors of CAR/VSV–treated mice compared to CAR/PBS–treated mice differed substantially. *k*-Means clustering with eight clusters highlighted differences in CD4 subpopulations, with clusters 3, 5, and 6 predominant within CAR/VSV–treated tumors (fig. S6, A and B). Moreover, multidimensional scaling analysis showed that CD4 T cells within each treatment group cluster together but are distinct from one another (fig. S6C). Cluster 6, defined by Thy1.1 expression, represents the tumor-infiltrating CD4 CAR. The predominance of CD4 CAR in CAR/VSV–treated mice suggests that VSV-IFN-β cotreatment also facilitates tumor infiltration of CD4 CAR, in addition to CD8 CAR. Clusters 3 and 5 both highly expressed T-bet, a transcription factor marker of T helper type 1 (T_H_1) CD4 T cells. T_H_1 cells mediate immunity against viral pathogens and secrete IFN-γ, which can potentiate CD8 T cell activity. Thus, part of the augmented effector phenotype of the TCR-primed CAR T cells could be explained by the effect of antiviral T_H_1 cells amplifying a CD8 response. In addition, cluster 6 expressed high levels of PD-1 and LAG3, both indicators of CD4 engagement and activation (fig. S6D). Overall, the picture of protein expression by intratumoral CD4 T cells supports T_H_1 polarization and CD4 engagement by VSV infection, which in turn may further activate transferred CD8 CAR T cells through cytokine or antigen-presenting cell (APC) interactions.

### Single-cell RNA sequencing reveals distinct tumor-infiltrating lymphocyte (TIL) population changes from CAR/VSV combination therapy

**Fig. 5.**
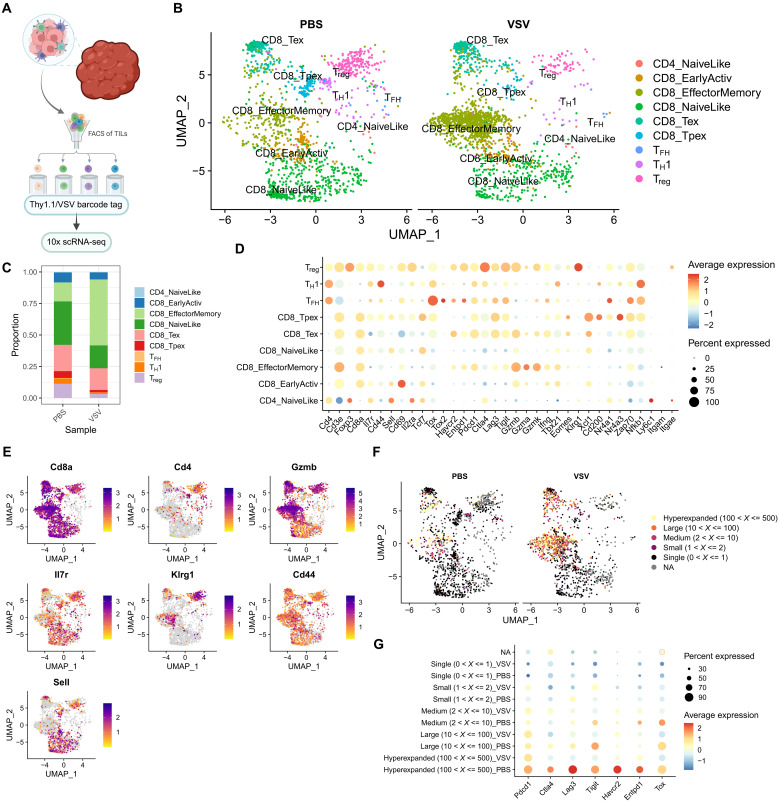
VSV promotes selective expansion of intratumoral effector memory CD8 T cells while enhancing the expression of effector-associated genes and sparing exhaustion. Single-cell RNA sequencing was performed on flow-sorted intratumoral T cells on day 16 of the experimental scheme outlined in Fig. 2A. (**A**) Schematic illustrating the flow sorting protocol before submitting for 10x Genomics single-cell RNA sequencing (scRNA-seq). FACS, fluorescence-activated cell sorting. (**B**) UMAP of overall intratumoral CD3+ T cell populations, clustered using Seurat and labeled with assistance from the ProjecTILs R package. T_FH_, T follicular helper cell. (**C**) Relative abundances of each labeled cluster by treatment condition. (**D**) Dot plot highlighting the expression of genes of interest within each labeled cluster. (**E**) UMAP plots colored by expression of genes of interest, revealing segregation of effector- and naïve/memory–associated markers. (**F**) TCR sequencing was used to identify regions of clonal expansion within phenotypic clusters, identifying the CD8 effector memory cluster as a focus for clonal expansion. NA, not applicable. (**G**) Dot plot focused on exhaustion-related genes to show targeted sparing of exhaustion within VSV-expanded clusters.

Using 10x Genomics single-cell RNA sequencing on tumor-infiltrating T cell populations (Fig. 5A), we observed that CAR/VSV cotreatment promoted the expansion of CD8 effector memory T cells (T_em_ cells) while reducing the proportion of CD4 regulatory T cells (T_reg_ cells) within the tumor-infiltrating lymphocyte (TIL) population (Fig. 5, B and C), consistent with our previous flow cytometric data. The CAR/VSV–expanded CD8 T_em_ cluster highly expressed cytotoxic and effector-associated markers such as *Gzma*, *Gzmb*, *Ifng*, *Cd44*, and *Klrg1*, in addition to low-level expression of *Nr4a3*, consistent with our Tocky data from Fig. 3 (Fig. 5, D and E). The CAR/PBS–predominant T_reg_ cluster displayed high levels of *Foxp3*, *Cd25*, *Lag3*, and *Ctla4* in accordance with traditional T_reg_ transcriptional profiles (*27*). Despite being abundant within tumors, minimal numbers of CAR T cells were Foxp3+ within our Tocky EGFRviii CAR T cell infusion product, implying potential T_reg_ induction within the tumor microenvironment or transient Foxp3 expression within the CAR population.

Analysis of TCR sequencing data highlighted clusters of clonal expansion, primarily within CD8 clusters, with higher levels of clonal expansion in the CAR/VSV treatment condition. Clonal expansion was associated with different exhaustion phenotypes between treatment conditions. Within the CAR/PBS group, expansion was associated with up-regulation of exhaustion markers including *Pdcd1*, *Ctla4*, *Lag3*, *Tigit*, *Havcr2*, *Tox*, and *Entpd1* (CD39), a marker more commonly used to denote tumor-reactive T cells. However, for expansion in the context of VSV, hyperexpanded and largely expanded cells seemed to uniquely evade exhaustion versus PBS-expanded CAR T cells, with comparatively lower expression of all evaluated exhaustion markers, which increases nevertheless with greater degrees of proliferation (Fig. 5, F and G). On the protein level, we confirmed that CAR T cell exhaustion increases with clonal expansion, as we observed both increased PD-1+LAG3+ expression on VSV N Tet+ CAR T cells and significantly greater levels of proliferation of TCR-primed CAR T cells 7 days after boosting with VSV-IFN-β compared to VSV N Tet− CAR T cells (fig. S7). However, this exhaustion-sparing phenotype suggests that CAR expansion in the context of virus activation/priming may preserve fitness over time and activity with reactivation compared to expansion in the context of potentially weaker, nonviral antigens.

### VSV promotes CD8 CAR cell TCR-mediated clonal expansion within effector populations

**Fig. 6.**
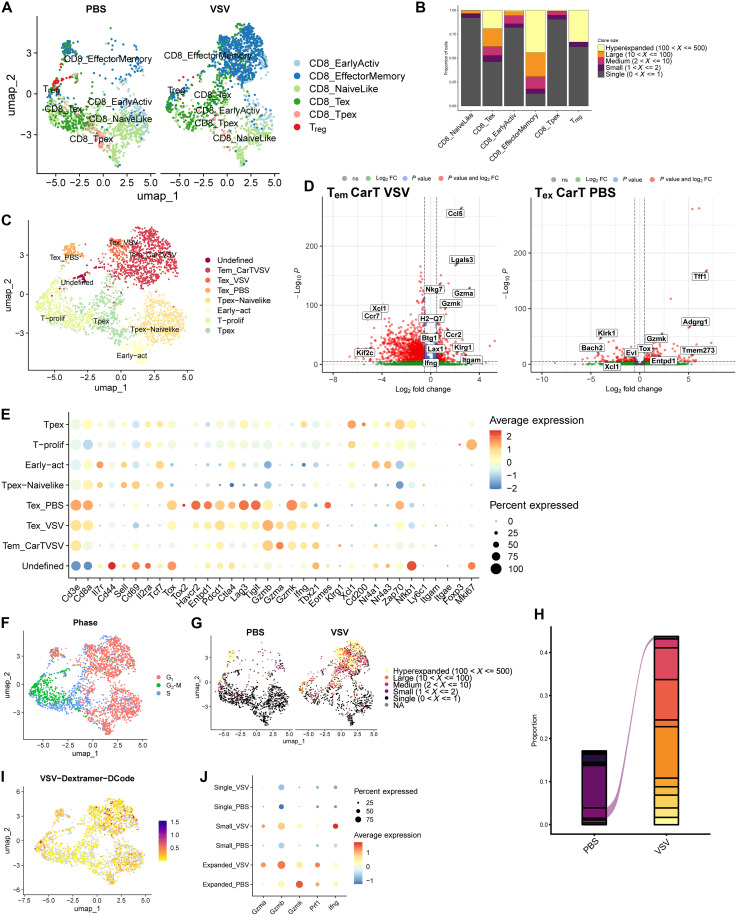
Tumor-infiltrating CD8 CAR T cells undergo clonal hyperexpansion against viral antigens. Single-cell RNA sequencing data were filtered for CD3+CD8+ CAR T cells, and clustering analyses were rerun. (**A**) UMAP of intratumoral CD3+CD8+ CAR T populations labeled with assistance from the ProjecTILs R package. (**B**) Abundance plot describing the level of clonal expansion within each CD8 CAR T cell cluster. (**C**) UMAP with relabeled clusters to consolidate original clusters on the basis of gene expression and activation state. (**D**) Volcano plot highlighting up- and down-regulated genes within the dominant expanded VSV and PBS clusters, T_em_ and T_ex_, respectively. FC, fold change. (**E**) Dot plot illustrating gene expression levels within each of the relabeled clusters from (C). (**F**) Cell cycle analysis was run and overlaid on a UMAP plot, with clusters featuring high levels of expansion predominantly in the G_1_ phase of the cell cycle. (**G**) TCR sequencing was used to identify clonally expanded T cells, with levels of clonal expansion defined by the number of identified clones. (**H**) The abundance plot of each clonotype within either the PBS or VSV condition illustrates minimal overlap in T cell clones. (**I**) A VSV N_52–59_ dextramer was used to identify antiviral CAR T cells. Positive events were identified primarily in VSV-expanded regions. (**J**) Differential gene expression of effector-associated proteins demonstrates increased expression among expanded T cell clones in the VSV condition versus PBS.

To better elucidate the transcriptomic diversity within CD8 CAR T cells between PBS- and VSV-IFN-β–treated mice, following dimensionality reduction and unsupervised clustering, samples were filtered for CD8 CAR T populations for focused evaluation. As seen previously in Fig. 5, CAR/VSV cotreatment greatly expanded the effector memory CAR T population and depleted a small *Foxp3*-high subset that seemed to be associated with PBS cotreatment (Fig. 6A). Between clusters, the effector memory CD8 CAR underwent the highest degree of expansion, with outsized populations of hyperexpanded and largely expanded CD8 clones. The small *Foxp3*-high subset also appeared to undergo expansion, potentially supporting the notion that a population of self-reactive CD8 CAR may be expanded with induced Foxp3 expression within the tumor microenvironment (Fig. 6B).

Using our initial clusters generated using ProjecTILs, we refined cluster labeling with additional gene expression and gene set enrichment analysis data (Fig. 6C). The T_em_ CarTVSV cluster comprised largely T cells with expanded TCRs by CDR3 sequence. These expanded T cells were found to highly express effector markers including *Gzma*, *Gzmk*, *Ifng*, and *Tbx21*, in addition to *Klrg1*. Adhesion- and chemotaxis-related markers such as *Ccl5*, *Ccr2*, and *Itgam* were also up-regulated in the VSV T_em_ cluster. Expanded terminally exhausted T cells associated with VSV (T_ex_ VSV) also expressed these cytotoxic markers, in addition to exhaustion-associated transcripts such as *Havcr2*, *Pdcd1*, *Lag3*, and *Tigit*, with little *Klrg1* expression. Because of the prevalence of cytotoxic, nonexhausted, hyperexpanded T cells within the T_em_ CarTVSV cluster, we propose that VSV-IFN-β may be promoting the formation of a pool of long-lived effector cells that have been characterized by other groups as KLRG1+ with high retention of cytotoxic proteins (Fig. 6, D and E) (*28*).

The primary cytotoxic cluster associated with PBS cotreatment represented terminally exhausted T cells (T_ex_ PBS). This cluster exhibited a high expression of exhaustion/dysfunction markers including *Tox*, *Havcr2*, *Pdcd1*, *Lag3*, and *Tigit*; low *Tbx21*; and high *Eomes*, with high *Zap70* (Fig. 6E). T-bet and Eomes were identified as two markers of CD8 T cell fate that were differentially expressed between PBS and VSV clusters. Although overexpression of T-bet has been shown to improve CD8 effector functions, high levels of Eomes are correlated with T cell exhaustion (*29*, *30*), highlighting differences between PBS and virus-mediated CAR T cell phenotype polarization. The defined clusters were also uniquely associated with varying stages in the cell cycle. The expanded clusters in both the PBS and VSV cotreatment conditions (T_ex_ PBS, T_em_ CarTVSV, and T_ex_ VSV) were predominant in the G_1_ phase, characterized by cellular growth and protein synthesis. The propensity of these CAR populations to remain in G_1_ suggests prior activation and potential to rapidly proliferate through a S-G_2_ dominant expansion phase, as well as a preference for cytotoxic effector pathways (Fig. 6F) (*31*, *32*).

As in Fig. 5, analysis of TCR sequences revealed a greater degree of hyperexpansion among CAR T cells administered with VSV compared to PBS, in addition to an increase in large and medium degrees of clonal expansion (Fig. 6G). Between expanded clones in the two conditions, there was very little overlap in TCR sequences, suggesting that VSV drove priming and proliferation of a unique set of TCRs, presumably viral-specific (Fig. 6H), as confirmed with the predominance of VSV N_52–59_ dextramer signal among hyper- or largely expanded clones in the CAR/VSV condition (Fig. 6I). These VSV-expanded clones seemed to adopt a particularly cytotoxic phenotype as well, overexpressing *Gzma*, *Gzmb*, and *Prf1* when compared to PBS-expanded clones (Fig. 6J).

### CD4 help enriches a distinct CAR T cell phenotype in the presence of VSV

**Fig. 7.**
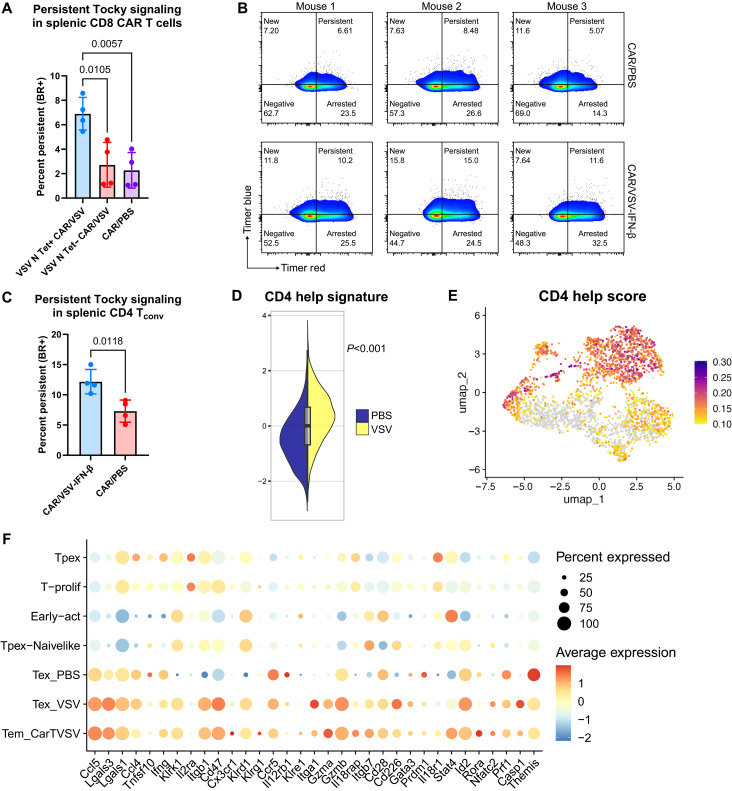
VSV-cotreated CD8 CAR T cells undergo increased interactions with CD4 T_conv_ cells, promoting cytotoxicity and tissue-homing capacity. (**A**) Increased expression of Tocky Timer protein in splenic CD8 CAR T cells, contrary to tumor CD8 CAR T cells, suggests increased CD8 T cell engagement in the spleen. (**B**) Representative flow plots of splenic CD4 Tocky Timer protein expression. (**C**) Increased expression of Tocky Timer protein in splenic CD4 T_conv_ cells implicates CD4 interactions within the spleen in augmenting CD8 CAR T cell function and tumor trafficking. (**D**) Referencing a publication from Ahrends *et al.* (*25*), the expression of their defined CD4 help signature within our CD8 CAR T cells was quantified from our single-cell RNA sequencing data. (**E**) The CD4-helped score was superimposed on a UMAP plot of CD3+CD8+ tumor-infiltrating CAR T cells and found to be concentrated in VSV-expanded and clonally expanded clusters. (**F**) Dot plot outlining the genes associated with a CD4 help phenotype, many of which appear to be up-regulated within VSV-associated clusters. Statistical significance was calculated using a one-way ANOVA with Tukey’s for multiple comparisons or an unpaired two-tailed *t* test for two-sample comparisons.

Whereas tumor-infiltrating CD8 CAR T cells were less activated via NFAT-Nr4a3 when cotreated with VSV compared to PBS, the opposite relationship was observed in the spleen. Within the spleen, VSV N_52–59_ Tet+ CD8 CAR T cells were more persistently engaged via Tocky than Tet− or PBS-cotreated CAR T cells (Fig. 7A). This suggests differences in potential binding interactions or antigen availability of the viral TCR antigen in the spleen, or systemic circulation, than in the tumor microenvironment following systemic viral delivery. Thus, using the Tocky profile of splenic CD4 T cells as a way to assess the activity of the “priming triad,” consisting of a CD8 T cell, an APC (commonly a dendritic cell), and a CD4 T cell (*26*), we found that, similar to the CD8 CAR T cells, CD4 conventional T cells (T_conv_ cells; non-Foxp3) in the spleen were also more persistently engaged, indicating increased CD4 T cell activation, potentially in the context of CD8 T cell priming (Fig. 7, B and C).

With the knowledge that splenic CD4 T cells were, at the very least, more active with VSV cotreatment compared to with no virus, we evaluated the degree of CD4 help given to CD8 T cells using a CD4 help signature defined within the transcriptome of CD8 T cells using vaccination with and without CD4 help via major histocompatibility complex class II–restricted helper epitopes (*25*). In that study, the transcriptome of CD4-helped CD8 T cells was distinct from those activated without help, with up-regulation of cytotoxic effector, migration, and prosurvival markers and down-regulation of co-inhibitory receptors such as *Pdcd1* and *Lag3*. By applying this signature to our single-cell RNA sequencing data, we observed that this CD4 help signature was significantly up-regulated in CD8 CAR T cells cotreated with VSV and that this signature was concentrated in clonally expanded CD8 T cell clusters, namely within the T_em_ CarTVSV cluster (Fig. 7, D and E). Evaluation of individual markers associated with the CD4 help phenotype demonstrated particularly strong associations with expanded VSV clusters (T_ex_ VSV and T_em_ CarTVSV). These VSV clusters up-regulated migration-associated markers such as *Ccl5*, *Ccl4*, *Itgb1*, *Cx3cr1*, and *Ccr5* and mediators of cytotoxicity and inflammation including *Tnfsf10*, *Ifng*, *Gzma*, *Gzmb*, *Prf1*, and *Casp1* (Fig. 7F). Up-regulation of this signature in CAR/VSV CD8 CAR highlights the role of VSV in engaging the immune microenvironment in part by recruiting CD4 T cells and APCs to more robustly activate CD8 T cell responses.

Depletion of CD4 T cells using antibody-based depletion confirmed the importance of CD4 T cell interactions in the overall generation of the TCR-primed CAR T cell population as evidenced by the decreased prevalence of VSV N_52–59_ Tet+ TCR-primed CAR T cells in both the spleen and tumor, with this decrease reaching significance in the tumor (fig. S8). The importance of these CD4 interactions could, to some degree, explain the improved CAR T cell functionality and antitumor efficacy of TCR-primed CAR T cells and underline the important role of TCR engagement in CAR T cell functions and phenotype.

## DISCUSSION

CAR T cell therapy, while having found substantial success in hematologic cancers, has faced many challenges in solid tumors (*2*). In addition, VSV alone as an OV has also yet to be US Food and Drug Administration–approved for solid tumors as a monotherapy, largely due to toxicity concerns and rapid immune-mediated clearance (*33*). Here, we show that, by generating a TCR-primed CAR T cell population through CAR/VSV combination therapy and subsequently boosting this population with systemic VSV, long-term therapy of mice could be achieved (Fig. 1) (*19*). This enhanced tumor therapy was associated with greater durability of the CAR T cell population in tumor-cured mice compared to those that succumbed to tumor. We then show that this combination of subtherapeutic intravenous VSV with CAR T cells markedly altered CAR T cell protein expression, signaling profiles, and transcriptomic signatures to promote effector phenotypes and CAR T cell function more capable of exerting long-term tumor control. Through study of differential CD8 T cell activation in the spleen and tumor of treated mice, in addition to comparative analyses of the CD8 CAR T transcriptome, we propose a potential mechanism through which CD8 CAR T cells become polarized to a hypereffector phenotype through VSV-mediated CD4 interactions in the spleen, facilitating significantly improved tumor infiltration and antitumor functions. Ultimately, this work thus highlights the potential benefits of in vivo TCR engagement via systemic therapies in polarizing and augmenting CAR T cell therapies.

We defined TCR-primed CAR T cells as having developed antiviral specificity against the immunodominant VSV N protein–derived epitope VSV N_52–59_ presented on H-2K^b^ (Fig. 2). We show that CAR T cells with the antiviral VSV N TCR proliferated to become a sizeable proportion—up to 50% within the tumor bed—of the total CAR population. In the presence of VSV-IFN-β, CAR T cells and CD8 T cells in general exhibited improved tumor infiltration. Uniquely, even within the same mice, CAR T cells that became TCR primed significantly down-regulated CD62L, CD127, and CD73, indicating polarization away from naïve or memory toward effector phenotypes. We also observed a notable bifurcation in CAR T cell function depending on the stimulating antigen (Fig. 2). Whereas stimulation with the CAR antigen drove granzyme B production in CD8 CAR T cells, engagement of the TCR antigen promoted a predominantly IFN-γ response, with the TCR-primed CAR T cells demonstrating superior functionality in response to both antigen types. While disparate T cell cytokine responses have been characterized between CAR and TCR engagement, this differential granzyme B and IFN-γ response represents a previously unidentified distinction between CAR and antiviral TCR engagement (*34*, *35*).

Using the transgenic Tocky mouse model as a reporter of T cell activation, we show that, despite carrying a second activating receptor, TCR-primed CAR T cells were paradoxically less persistently engaged through either activating receptor when measured by expression of the Timer protein at 7 days post–viral administration (Fig. 3). We hypothesize that the process of TCR priming and/or the presence of activating TCR signals from presentation of viral antigens could potentiate the CAR T cell to promote “fast-off” binding interactions, leading to lower duration T cell activation and decreased expression of Nr4a3, as reflected by diminished Timer protein expression observed in TCR-primed CAR T cells. In our model, this relatively low-level Tocky signal within the TCR-primed CAR T cells could also be explained by early clonal expansion of the CAR T cells in response to VSV antigens, leading to acute T cell activation and signaling, with Timer protein being lost as the T cells proliferate in response to transient viral antigens. However, we observed this difference in persistent signaling within the tumor bed, an environment with ample CAR antigen and activating ligands. Thus, the difference in persistent Tocky signaling within the tumor between TCR-primed and non–TCR-primed CAR T cells could be related to prior priming of a high-affinity TCR continuing to serve as a potentiator or agonist of CAR signaling, lowering the threshold of activation of CAR signaling for effector functions and resulting in continually low persistent engagement of the CAR itself. This hypothesis is supported by both the amplified effector functions upon TCR or CAR stimulation ex vivo of the TCR-primed CAR (Fig. 2) and the correlation of improved tumor control with less persistently engaged CAR T cells in the tumor (Fig. 3). In contrast, the non–TCR-primed CAR T cells were more persistently engaged, presumably requiring a higher threshold of downstream activation to exert cytotoxic functions and proliferate, which may contribute to an increased state of exhaustion/dysfunction. This concept of “fast-off” binding, characterized by a high *K*_on_ (on-rate constant) and *K*_off_ (off-rate constant), has been demonstrated in CAR T cells to maintain tumor killing while avoiding T cell exhaustion (*36*). Similarly, we observed a strong correlation between antitumor therapy and low levels of persistent signaling in CAR T cells, consistent with this hypothesis that viral antigen TCR engagement in CAR T cells alters the nature of the CAR interaction with target cells to a more rapid “fast-off” phenotype (Fig. 3). In addition, Nr4a3 expression in CAR T cells has been shown to promote T cell exhaustion and dampen antitumor functions, consistent with our findings that TCR-primed CAR T cells display diminished NFAT signaling (*37*). Therefore, our studies of anti-VSV TCR-primed CAR T cells show that CAR T cells can regulate activating signals through endogenous TCR engagement while maintaining or enhancing effector functions and gene profiles.

In this respect, the CyTOF data in Fig. 4 revealed significantly increased levels of CD11c, T-bet, iNOS, Fas, IRF4, and BATF on CD8 CAR T cells cotreated with VSV compared to PBS cotreatment. CD11c expression on CD8 T cells has been associated with increased antitumor activity, granzyme B and cytokine production, and degranulation in patients with hepatocellular carcinoma, and in a murine model of lymphocytic choriomeningitis virus infection, CD11c expression on CD8 T cells correlated with cytotoxic capacity (*22*, *38*). Knockout of IRF4 and BATF in murine models demonstrated a loss of effector functions and effector fate commitment in CD8 T cells, highlighting the role of these transcription factors in deciding and maintaining T cell effector fates (*39*, *40*). In addition, Fas, conventionally involved in regulating apoptotic pathways, was up-regulated on CD8 T cells upon dendritic cell interactions and was implicated in maintaining CD8 expression upon repeated TCR stimulation (*41*). Specifically, in CAR T cells, overexpression of T-bet was recently correlated with improved antitumor functions without a subsequent loss in persistence (*29*). Overall, the protein expression profile uncovered by our high-parameter CyTOF analysis showed expansion of a CAR T cell population characterized by up-regulation of effector-associated markers. We thus refer to these VSV-polarized CD8 CAR T cells as hypereffector CAR T cells. The exaggerated effector profile of these CAR T cells lends support to the beneficial effects that can result from TCR engagement on CAR T cell therapies.

Furthermore, the focal expression of CD69 and CD25 highlighted in the CyTOF data indicates small clusters of unique phenotypes of CD8 CAR T cells. As mice were treated with VSV 7 days before CyTOF analysis, it is unlikely that the CD8 T cells in the CD69+ resident memory–like cluster were acutely activated. Therefore, the high CD69 expression could indicate the early formation of a resident memory pool of CAR T cells (*42*). High CD25 expression in CD8 T cells after TCR activation is correlated with shorter-lived effector cells, whereas lower CD25 expression in expanded CD8 T cells is more correlated with longer-lived memory T cells (*43*). Thus, the CD25+ CAR cluster could represent a population of especially short-lived effector CAR T cells with high cytotoxic capacity.

We also show here marked VSV-mediated clonal expansion of CAR T cells using single-cell RNA sequencing of TILs. Infused CAR T cell products in humans consist of a polyclonal pool of T cells; in those settings, clonal kinetics have been tracked postinfusion to delineate distinct in vivo kinetics depending on CAR T clonotype (*44*). However, TCR-mediated CAR T clonal expansion against a known antigen is, we believe, a previously unexplored finding within the field of CAR T cell therapy. CD8 CAR T cells expanded with VSV were spared an exhausted fate compared to their PBS-expanded counterparts, down-regulating the expression of *Pdcd1*, *Ctla4*, *Lag3*, and *Havcr2*. Separately, these VSV-expanded CARs up-regulated the expression of *Gzmb*, *Gzma*, *Prf1*, and *Ifng*, consistent with their enhanced acquisition of effector properties. These effects can be in part explained through increased interactions with CD4 T cells in the spleen, highlighted by increased splenic CD4 and CD8 T cell engagement. This CD4 help promotes the expression of CD8 effector molecules and improves CD8 tissue invasiveness, both of which we observed in this study (*25*). Beyond the phenotype, we showed that CD4 T cells are essential for the generation of TCR-primed CAR T cells themselves. Interactions with CD4 T cells and potential APCs appear to particularly influence the ability of adoptively transferred CD8 CAR T cells to expand in response to a TCR antigen and therefore form a subpopulation of antiviral, TCR-primed CAR T cells. Thus, our data here show that CAR T cell TCR engagement, through exposure to highly immunodominant foreign viral antigens in vivo, is an alternative, clinically relevant, and potent route through which CAR T cells can be selectively expanded, maintained, and polarized in vivo.

Although our experiments here exclusively used a tetramer against VSV N_52–59_ to detect antiviral CD8 T cells, many other epitopes of VSV are likely to have primed both endogenous and CAR T cell populations and may have resulted in different phenotypes of TCR-primed T cells. In addition, the contribution of epitope spreading due to the activity of the OV in the TCR priming of CAR T cells is not clear nor is the antigen hierarchy regarding the affinity and avidity of viral versus tumor antigens. However, the predominance of the TCR priming of CAR T cells by immunodominant viral epitopes compared to the largely immunosubdominant self/near-self tumor-associated antigens makes it unlikely that these subdominant TCR-primed CAR T cells make up a major population (*45*). It is thus probable that the CAR T cell antiviral response is outcompeting clonal expansion against tumor antigens, but given the demonstrated utility of a known, high-affinity TCR, this is likely a favorable outcome (*46*). Studies to investigate the importance of the strength (affinity/avidity) of the CAR T cell TCR signal in the generation of therapeutically active TCR-primed CAR T cells are currently underway. In addition, previous studies have confirmed that human CAR T cells can be engaged through their endogenous TCR, and both CAR T cell and VSV therapies have been trialed separately in humans against solid tumors and for vaccine purposes, respectively (*47*, *48*). Studies to show the development of TCR-primed human CAR T cells are currently underway in our laboratory as a prelude to translating the combination of CAR T cells and subtherapeutic VSV to humans for generating hyperfunctional subsets of CAR T cells in patients.

Our studies here add a significant value to previous reports that have explored the beneficial effects of functional activation of the endogenous TCR of CAR T cells. With the hope of using a preexisting population of endogenous antiviral T cells, CAR T cells have been generated from human peripheral blood postexpansion with Varicella Zoster virus or cytomegalovirus peptide mixes. These antiviral CAR T cells were able to eliminate both CAR and TCR targets and expand in response to TCR stimulation (*10*, *11*). Using mouse models with transgenic TCRs, for example, from OT-1 or pMEL mice, to generate CAR T cells with known TCR specificity, dual CAR and TCR signaling was shown to generate optimal therapy. Consistent with our findings here, TCR stimulation was effective at driving CAR T cell proliferation, and in the case of OT-1 CAR T cells, signaling through the CAR and TCR was modeled to illustrate a diversity in CAR-TCR cross-talk across varying TCR signal strengths (*12*, *49*). Our data expand on these results in that they present a clinically implementable protocol in which CAR T cells can be combined with OV therapy to generate a population of TCR-primed CAR T cells with distinct, clinically beneficial properties of enhanced persistence and boost-able activity. In addition, our results suggest a novel utility for systemically administered oncolytic virotherapy. Thus far, systemic delivery of OV has proved challenging in the face of antiviral neutralizing antibodies, proper tumor-specific dosing, and patient safety (*33*, *50*). Our laboratory has shown that intravenous VSV can access tumors at low frequencies and, at least in the B16/C57BL/6 model, is therapeutically largely ineffective (*19*). We have also reported that direct intratumoral injection of the highly inflammatory VSV can lead to attrition of tumor-infiltrating CAR T cells because of high levels of type I IFN (*20*). In contrast to these challenges, our data show here that, as well as potentially providing direct oncolytic and immunomodulatory benefits, intravenous administration of OV in combination with CAR T cells can be highly therapeutically effective through its capacity to reshape and boost CAR T cell activity. Given the acute timeline of VSV infectivity in vivo, we were able to use transient VSV infection to reshape CAR T cell responses against CAR antigens, which are retained on tumor cells, indirectly using a short-lived TCR antigen to augment the function and phenotype of CAR T cells against their more stable target (*51*, *52*).

In summary, we show here the value of activation of the endogenous TCR in focusing and augmenting CAR T cell phenotype and function. CAR T cells whose TCRs were engaged and who became primed against a viral antigen—TCR-primed—acquired a unique hypereffector phenotype, defined by increased expression of effector-associated proteins like CD11c, granzyme B, IFN-γ, T-bet, and CD44. Despite carrying two activating receptors, TCR-primed CAR T cells existed in a less activated state compared to traditional, non–TCR-primed CAR T cells via decreased Nr4a3 expression. CAR T cells cotreated with VSV also underwent clonal expansion, amplifying a separate clonal repertoire from PBS-cotreated CAR. Thus, our data support the exploitation of the TCR of CAR T cells as a tool through which CAR T cells can be expanded in vivo and differentiated toward new potent antitumor phenotypes, properties that could have significant impacts on patient outcomes in the fight of CAR T cells against solid tumors.

## MATERIALS AND METHODS

### Study design

These experiments were designed to interrogate the phenotype of a population of antigen-specific CAR T cells that arise upon cotreatment with the OV VSV in the setting of a solid tumor. We previously marked this population of anti-VSV N_52–59_ TCR-primed CAR T cells and their ability to mediate curative therapy in murine melanoma and glioma solid tumor models. These particular experiments focus on understanding the effector versus memory phenotype of these CAR T cells and the effect of OV-CAR combination on CAR T activation and function. Immunocompetent mouse studies were performed to generate and study TCR-primed CAR T cells and were supplemented with a series of ex vivo studies and high-dimensional protein and transcriptomic analyses. Tetramer staining and peptide restimulation followed by measurement of intracellular cytokines were carried out to determine viral specificity. The investigators were not blinded during in vivo studies or related analyses. In vivo numbers were determined on the basis of results from pilot experiments, previous studies, and published data. The *n* values and statistical methods are outlined in the figure legends and the “Statistical analysis” section.

### Cell lines and viruses

B16 murine melanoma cells, baby hamster kidney (BHK) cells, and 293T human embryonic kidney cells were originally purchased from the American Type Culture Collection and grown in Dulbecco’s modified Eagle’s medium (Corning) + 10% fetal bovine serum (FBS; Gibco). Cells were regularly tested for mycoplasma using the American Type Culture Collection Universal Mycoplasma Detection Kit. The B16EGFRviii cell line was generated by lentiviral transduction of B16F10 cells with a self-inactivating lentiviral vector encoding the murine EGFRviii protein containing a 500–amino acid deletion in the intracellular domain. A polyclonal population of EGFRviii-expressing B16 was maintained in puromycin (1.25 μg/ml; Sigma-Aldrich).

VSV expressing murine IFN-β was rescued from the pXN2 cDNA plasmid using the established reverse genetics system in BHK cells, as described previously (*53*). For this protocol, BHK cells were infected with MVA-T7 at a multiplicity of infection (MOI) of 1. After 1 hour, cells were transfected with the pVSV-XN2 genomic VSV plasmid, pBluescript (pBS)–encoding VSV N, pBS-encoding VSV P, and pBS-encoding VSV L using Fugene6 according to the manufacturer’s recommendations. After 48 hours, the supernatant was collected and clarified by passing through a 0.22-μm filter to remove cell debris. Viral stocks were purified through a 10% sucrose cushion. Viral titers were determined using a plaque assay on BHK cells.

### Mice

Female C57BL/6 mice (strain no. 000664) were obtained from the Jackson Laboratory. All mice were received at 4 to 8 weeks of age and housed in a biosafety level 2 biohazard facility. Experimental mice were cohoused and exposed to a 12-hour light/12-hour dark cycle with unrestricted access to food and water. The ambient temperature was kept between 20.6° and 26.1°C, and the room humidity ranged from 30 to 70%. All animal studies were done in compliance with and approved by the Institutional Animal Care and Use Committee at Mayo Clinic (protocol A00007023).

### Murine CAR T cell generation

Murine CAR T cells were generated from splenocytes isolated from C57BL/6 mice aged 6 to 12 weeks. Splenocytes were processed into a single-cell suspension and cultured in RPMI (Corning) containing 10% FBS, 50 μM 2-mercaptoethanol (Sigma-Aldrich), 1% penicillin-streptomycin (Corning), 1% nonessential amino acids (Corning), 1% sodium pyruvate (Corning), human interleukin-2 (IL-2; 50 U/ml; Novartis), and concanavalin A (2.5 μg/ml; Sigma-Aldrich). The retroviral supernatant was produced from 293T cells transfected with the MSGV1 retroviral plasmid along with the plasmid pCL Eco (Takara) encoding Gag, Pol, and an ecotropic envelope. The MSGV1 retroviral construct contains a third-generation EGFRviii CAR including CD28, 4-1BB, and CD3ζ moieties with an scFv derived from the human monoclonal antibody 139 and the marker Thy1.1 (CD90.1). T cells were transduced using the retroviral supernatant on RetroNectin-coated plates (Takara) after 2 days of culture. Cells were split 1 day after transduction, maintained in IL-2 in culture, and used for in vivo injections on day 4 or 5. Thy1.1 expression was used to identify transduced CAR T cells.

### In vivo studies

Mice were challenged subcutaneously with 2 × 10^5^ B16EGFRviii cells in 100 μl of sterile saline (Hospira). For experiments requiring irradiation, mice bearing tumors received 5–gray (Gy) total body irradiation 24 hours before CAR therapy. Mice were treated with 1 × 10^7^ EGFRviii CAR T cells in 100 μl of sterile saline intravenously on day 4 or 7, depending on the experiment. Mice receiving virus were treated intravenously with 1 × 10^7^ plaque-forming units (PFU) of VSV-IFN-β in 100 μl of sterile saline. All intravenous injections were administered in the tail vein. Intratumoral VSV-IFN-β was administered at a dose of 1 × 10^8^ PFU in 50 μl of sterile saline. For experiments requiring CD4 depletion, a monoclonal anti-mouse CD4 antibody (Bio X Cell, no. BE0003-1; clone GK1.5) was administered intraperitoneally at a dose of 500 or 250 μg per mouse. Tumors were measured using calipers three times per week, and mice were euthanized using CO_2_ when tumors reached 1.0 cm in diameter or the study end point. For survival studies, the end point was reached when the tumor reached 1 cm in diameter.

### In vitro loading of CAR T cells

CAR T cells prepared as described above were collected on day 4 or 5 of the production protocol and pelleted and washed twice with PBS at 4°C. CAR T cells were then resuspended in PBS with VSV-IFN-β at a MOI of 1 and incubated at 4°C for 1 hour. The CAR T cells with VSV were then washed two or three times with PBS and resuspended in sterile saline for injection.

### qRT-PCR of CAR T cells in the periphery

Spleens of mice treated with UTDs or loaded/nonloaded EGFRviii CAR T cells with or without a VSV boost were harvested at the mouse end point (tumor reaching 1 cm in diameter in any dimension). These spleens were processed to form single-cell suspensions, and RNA was isolated from splenocytes using the Qiagen RNeasy Plus Mini Kit (no. 74134). From isolated RNA, cDNA was synthesized using the Roche Transcriptor First Strand cDNA Synthesis Kit (no. 04896866001). This cDNA was then used as template DNA for a PCR reaction with primers flanking the psi region of the CAR retroviral vector for CAR-specific detection. The Roche LightCycler 480 SYBR Green I Master kit (no. 04707516001) was used to perform the PCR reaction in a Roche LightCycler 480 Instrument II for quantification of qRT-PCR.

### Ex vivo coculture/restimulation assays

Splenocytes from mice treated with CAR T cells with or without VSV-IFN-β were isolated, processed into single-cell suspensions, and counted. Splenocytes (1 × 10^6^) were cultured with 1 × 10^5^ B16F10 or B16EGFRviii with or without VSV N_52–59_ peptide (1 μg/ml). Cells were then cocultured overnight, followed by addition of GolgiPlug (1:1000 dilution; BD Biosciences) for 4 hours. Intracellular staining was performed on cells incubated with GolgiPlug using a cellular fixation/permeabilization kit (BD). Cocultures were also flow stained with extracellular and intracellular fluorochrome-conjugated antibodies, as described in the following “Flow cytometry” section.

### Flow cytometry

Flow cytometry was performed on cultured cells and single-cell suspensions from dissected spleens, blood, and tumors. Before processing, tumors were weighed then treated with Liberase TL (Roche), deoxyribonuclease I (Roche), and 0.25% trypsin (Corning) in PBS for 30 min at 37°C. Blood was collected by submandibular vein bleed. All dissected tissues underwent red blood cell lysis with Ammonium-Chloride-Potassium Lysis Buffer before staining. Samples were either run live (resuspended in PBS + 2% FBS) or fixed in 4% formaldehyde. The Cytek Aurora spectral flow cytometer and SpectroFlo analysis software in the Mayo Clinic Flow Cytometry Core were used to acquire data and perform single-color compensation and spectral unmixing. Data were analyzed using FlowJo version 10.10.

Mouse cells were stained with fluorochrome-conjugated antibodies against the following antigens: CD3 (BioLegend, no. 100216; clone 17A2; 1:200 dilution), CD8b.2 (BioLegend, nos. 140418, 140416, and 140404; clone 53-5.8, 1:200 dilution), CD4 (BioLegend, no. 100537; clone RM4-5; 1:200 dilution), Thy1.1 (BioLegend, no. 202526; clone OX-7; 1:500 dilution), CD44 (BioLegend, no. 103059; clone IM7; 1:400 dilution), CD69 (BioLegend, no. 104537, clone H1.2F3; 1:400 dilution), CD39 (BioLegend, no. 143812; clone Duha59; 1:1000 dilution), CD73 (BioLegend, no. 127215; clone TY/11.8; 1:400 dilution), CD62L (BioLegend, no. 104418; clone MEL-14; 1:400 dilution), CD127 (BioLegend, no. 135008; clone A7R34; 1:200 dilution), PD-1 (BioLegend, no 135252; clone 29F.1A12; 1:200 dilution), and LAG3 (BioLegend, no. 125262; clone C9B7W; 1:200 dilution). Zombie fixable live-dead viability dye (BioLegend, no. 423106; 1:1500 dilution) was used to determine cell viability. The CellTrace Violet Cell Proliferation Kit (Invitrogen, no. C34557) was used to assess CD8 T cell proliferation.

To determine TCR specificity, cells were also stained with the H-2K^b^ VSV N_52–59_ RGYVYQGL BV421-labeled tetramer at a dilution of 1:100. The tetramer was obtained from the National Institutes of Health Tetramer Core Facility.

Intracellular staining was performed on cells cultured overnight with either B16 tumor cells, VSV N peptide, or both, followed by a 4-hour incubation with GolgiPlug (1:1000 dilution; BD Biosciences). Intracellular staining was performed on cells incubated with GolgiPlug using a cellular fixation/permeabilization kit (BD) and the following fluorochrome-conjugated antibodies: IFN-γ (BioLegend, no. 505806; clone XMG1.2; 1:100 dilution) and granzyme B (BioLegend, no. 372208; clone QA16A02; 1:100 dilution).

### Timer of cell kinetics and activity (Tocky)

The Tocky transgenic mouse was obtained from M. Ono’s lab at Imperial College London. Tocky CAR T cells were produced as described in the “Murine CAR T cell generation” section. For Tocky studies, Tocky CAR T cells were used in place of C57BL/6 CAR T cells, and Tocky mice were used in place of C57BL/6 experimental mice. In the Tocky model, Timer protein is first expressed as a blue fluorescent protein (*t*_1/2_ of 7 hours) but then spontaneously degrades to a red fluorescent protein (*t*_1/2_ of 20 hours). This allows for analysis of the timing, frequency, and strength of TCR signaling via the intensity and proportion of intracellular blue versus red protein. The activation state of Tocky T cells is divided into three categories: new (mostly blue Timer), persistent (mix of blue and red Timer), and arrested (mostly red Timer). New describes a T cell that was recently activated without sustained TCR engagement, persistent describes a cell with sustained engagement, and arrested represents a T cell that was stimulated previously but is no longer experiencing TCR engagement or signal transduction. The Tocky Timer-blue and Timer-red fluorescence was detected in the Pacific Blue and PE-Texas Red channels of a Cytek Aurora, respectively. Transgenic GFP (green fluorescent protein) expression was used to detect Foxp3. For in vitro Tocky experiments, untransduced and CAR T cells were cultured in complete media described in the “Murine CAR T cell generation” section above, with IL-2 (50 U/ml). T cells were cultured in vitro for 24 hours in plain IL-2–containing media, media supplemented with concanavalin A (2.5 or 1.25 μg/ml; Sigma-Aldrich), or media supplemented with CD3/CD28–activating beads at a 1:1 cell:bead ratio (Gibco, no. 11456D). For in vitro coculture experiments, 5 × 10^5^ T cells were incubated with 1 × 10^5^ B16 for 24 hours. For in vivo experiments, tissue processing, extracellular flow staining, and ex vivo stimulation were performed as described. For Tocky analyses, compensated gated populations of interest were exported as csv files from FlowJo and imported into R for angle transformation. Tocky locus analysis was run as developed and described by M. Ono (*21*).

### CyTOF

Tumors isolated on day 16 from mice receiving CAR T cells with either VSV-IFN-β or PBS were processed to a single-cell suspension and underwent red blood cell lysis. All samples were enriched for CD45+ leukocytes using magnetic bead isolation with CD45 (TIL) microbeads (Miltenyi, no. 130-110-618). CD45-enriched tumor samples were submitted to the Mayo Clinic Immune Monitoring Core for CyTOF preparation and data collection. Before data analysis, raw fcs files were filtered to remove aggregates, debris, doublets, and dead cells. Filtered data were further gated for specific immune cell populations and exported for clustering analyses. CyTOF clustering and expression analyses were performed using R version 4.4.0 following a CyTOF workflow developed by Nowicka *et al.* (*54*) that uses the HDCytoData and CATALYST packages.

### Single-cell RNA sequencing

Tumors isolated on day 16 from mice receiving CAR T cells with either VSV-IFN-β or PBS were processed to a single-cell suspension following red blood cell lysis and pooled. Samples were stained using fluorochrome-conjugated antibodies against CD3, CD4, CD8, and Thy1.1 and the Zombie fixable live-dead viability dye. Cells were also stained with a PE (phycoerythrin)–conjugated dCODE H-2K^b^ VSV N_52–59_ dextramer to detect VSV N–specific T cells. A Thy1.1 TotalSeq antibody was used to barcode label Thy1.1+ CAR T cells for CITE-Seq (cellular indexing of transcriptomes and epitopes by sequencing) (BioLegend, no. 202549; clone OX-7; 1:200 dilution). CD3+ T cells were isolated by cell sorting on a FACS Aria II, and a mix of dextramer and Thy1.1-positively and Thy1.1-negatively expressing T cells was submitted to the Mayo Clinic Genome Analysis Core for single-cell RNA sequencing including gene expression, CITE-Seq, and TCR sequencing using a Chromium Controller platform (10x Genomics) with a target capture rate of 10,000 individual cells per sample.

### Single-cell RNA sequencing data processing

Raw data were aligned and preprocessed using CellRanger to align reads, assemble V(D)J contigs, identify true cell events, and demultiplex the data. Following preprocessing and initial quality control, the data were subjected to secondary quality control and primary analysis using the Seurat pipeline. Cells were identified on the basis of abundance and novelty of genes, and genes were filtered on the basis of expression level. The effects of cell cycle and mitochondrial gene expression were accounted for because of their potential impacts on downstream clustering. Seurat was used to scale and normalize the data, after which data were subjected to dimensionality reduction and clustering to generate principal components analysis and uniform manifold approximation and projection (UMAP) plots for data visualization. If necessary, data were integrated to reduce batch or condition effects on clustering using gene anchors to cluster cells of the same type together. All secondary analyses were conducted using R version 4.4.0.

### Statistical analysis

Data processing was performed in Microsoft Excel. Graphing and statistical analyses were performed with GraphPad Prism 10 software (GraphPad). Single comparisons were made using unpaired two-tailed *t* tests. Multiple comparisons were analyzed using one-way analyses of variance (ANOVAs) followed up with a Tukey’s post hoc multiple comparison test. Survival data were assessed using a log-rank Mantel-Cox test with Bonferroni correction for multiple comparisons. Grouped data are plotted as the means ± SD.
