# Supplementary material for: Engagement of the TCR against an oncolytic virus generates a population of effector CAR T cells with potent antitumor activity
Source: Sci Adv. 2026 Jun 5;12(23):eaef5331. doi: 10.1126/sciadv.aef5331 (PMC13240226; doi:10.1126/sciadv.aef5331)
Supplement: Supplementary file 1 — Figs. S1 to S8 [file sciadv.aef5331_sm.pdf]

Science Advances
AAAS

Supplementary Materials for

Engagement of the TCR against an oncolytic virus generates a population of effector CAR T cells with potent antitumor activity

Olivia Liseth *et al.*

Corresponding author: Richard Vile, vile.richard@mayo.edu

Sci. Adv. **12**, eaef5331 (2026)
DOI: 10.1126/sciadv.aef5331

This PDF file includes:

Figs. S1 to S8

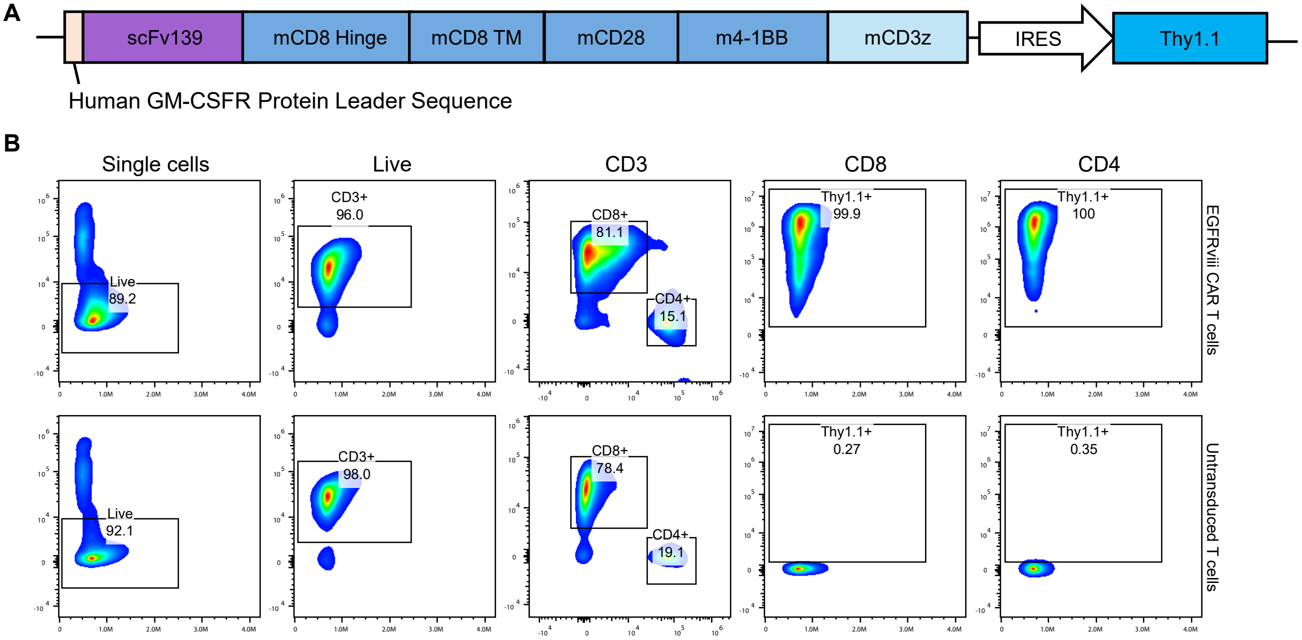

Supplemental Figure 1. Schematic of our EGFRviii CAR T cell construct and representative CAR T cell transduction efficiencies. A) The CAR construct is a MSGV1 retrovirus encoding the human GMCSFR leader sequence for membrane homing, the antibody scFv against EGFRviii, murine CD8 hinge and transmembrane regions, and murine CD28, 4-1BB, and CD3ζ intracellular signaling domains. Thy1.1 (CD90.1) is encoded following an IRES. B) Representative flow scheme detailing typical cell viability, T cell populations, and transduction efficiency following murine CAR T production (on day 4 or 5 post splenocyte harvest).

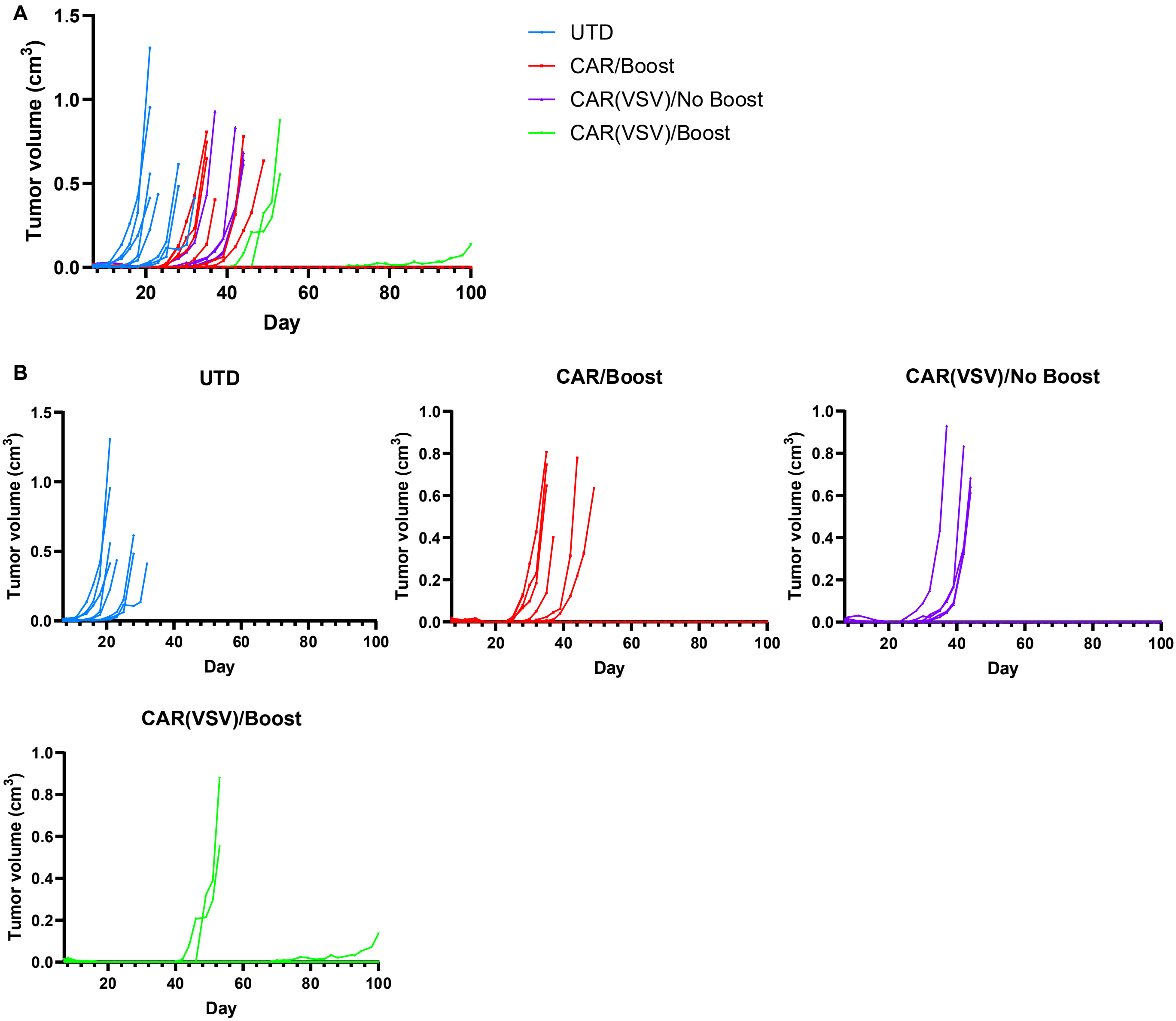

Supplemental Figure 2. Tumor growth curves for survival experiment with loaded CAR plus boost from Figure 1. A, B) Mice bearing subcutaneous B16EGFRviii tumors were treated with either 10^7 EGFRviii CAR T cells loaded with VSV-IFNβ (MOI 1) at 4C for 1 hour (CAR(VSV)), 10^7 EGFRviii CAR T cells, or 10^7 untransduced T cells (UTDs) on day 7 post tumor implantation and then boosted with either PBS or 10^7 pfu VSV-IFNβ on day 14. Calipers were used to measure tumor dimensions.

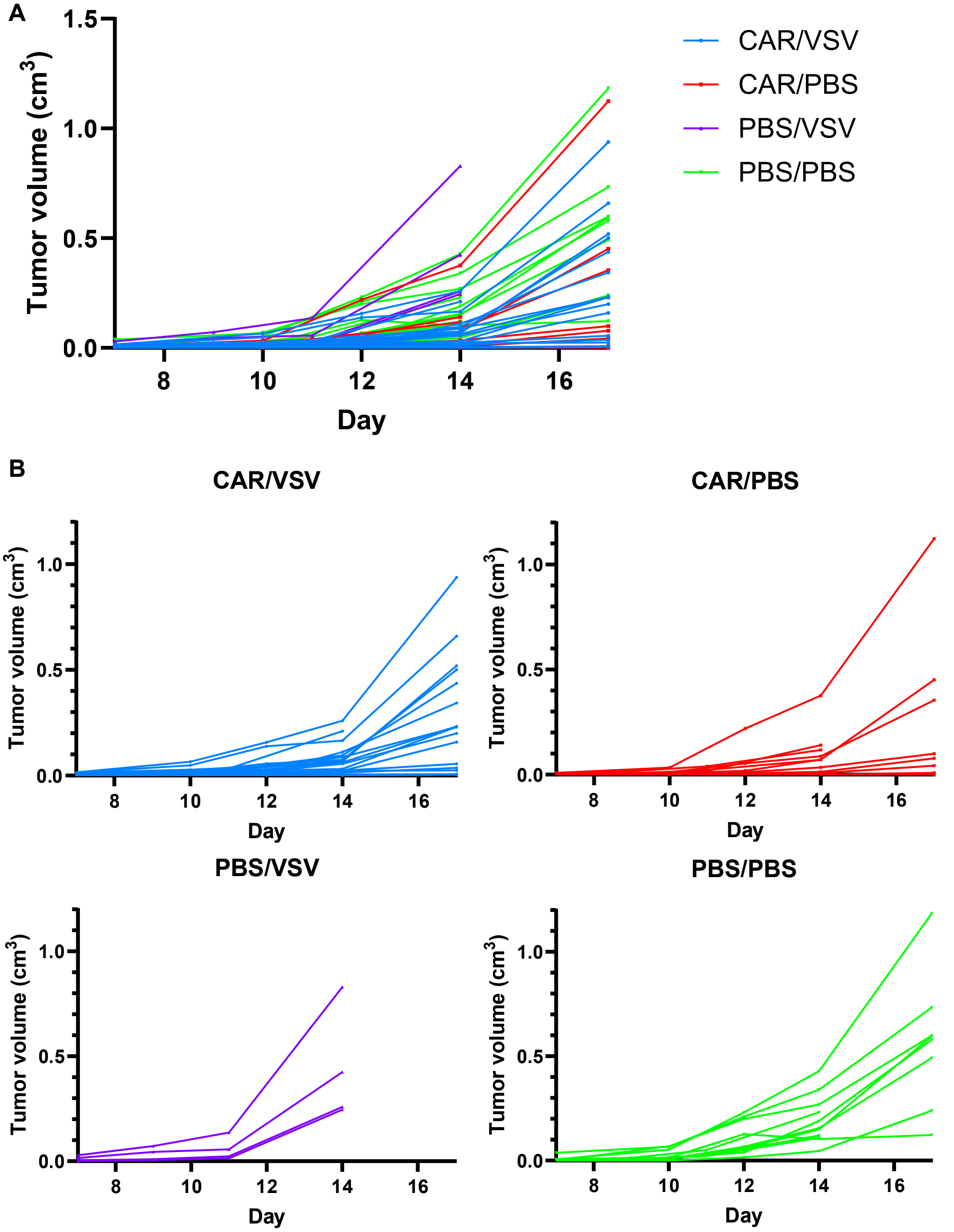

Supplemental Figure 3. Tumor growth curves for CAR/VSV sequential treatment from Figures 2-7. A, B) C57BL/6 mice bearing subcutaneous B16EGFRviii tumors were lymphodepleted on day 3 with 5 Gy total body radiation, treated on day 4 with 10^7 EGFRviii CAR T cells or PBS, and on day 9 with PBS or with VSV-mIFNβ intravenously (i.v.) (10^7 pfu). Calipers were used to measure tumor dimensions. Data are compiled from 3 independent experiments.

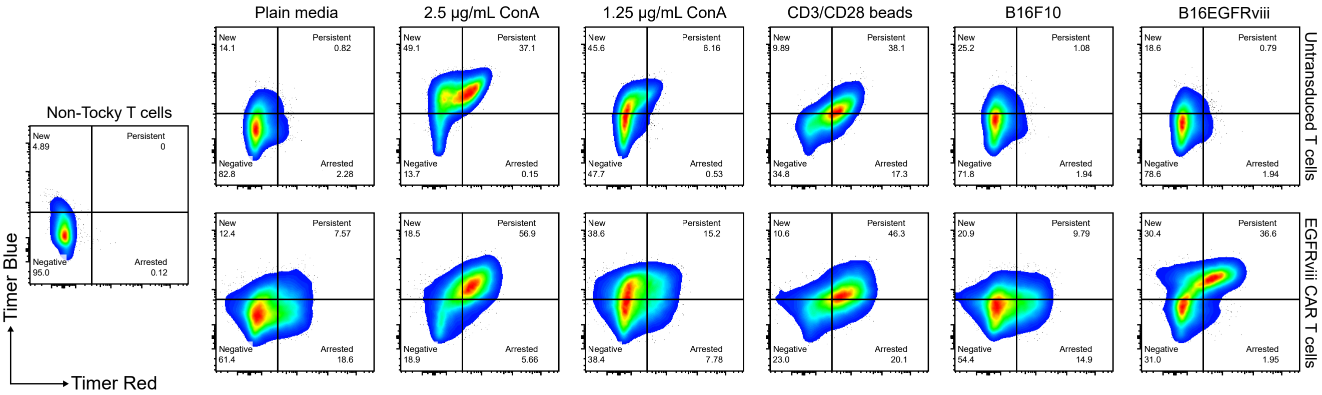

Supplemental Figure 4. Activation through both a CAR and a TCR results in Timer protein expression in the Tocky model. Signaling through both the TCR (via addition of concanavalin A or CD3/CD28 activating beads to T cell culture) and CAR (via CAR T cell coculture with B16EGFRviii) induces expression of Timer protein, seen in increased frequency of Blue+ (New), Blue+Red+ (Persistent), and Red+ (Arrested) T cells. EGFRviii CAR T cells or non-CAR untransduced mouse T cells were cultured in plain media, media containing concanavalin A (ConA) at 2.5 μg/mL or 1.25 μg/mL, or CD3/CD8 activating beads at 1:1 beads:T cell, or cocultured with B16F10 or B16EGFRviii at E:T of 5:1 for 24 hours. Cells were harvested and stained for T cell and CAR markers. Data shown are representative for n=3 technical replicates of live CD3+CD8+ T cells.

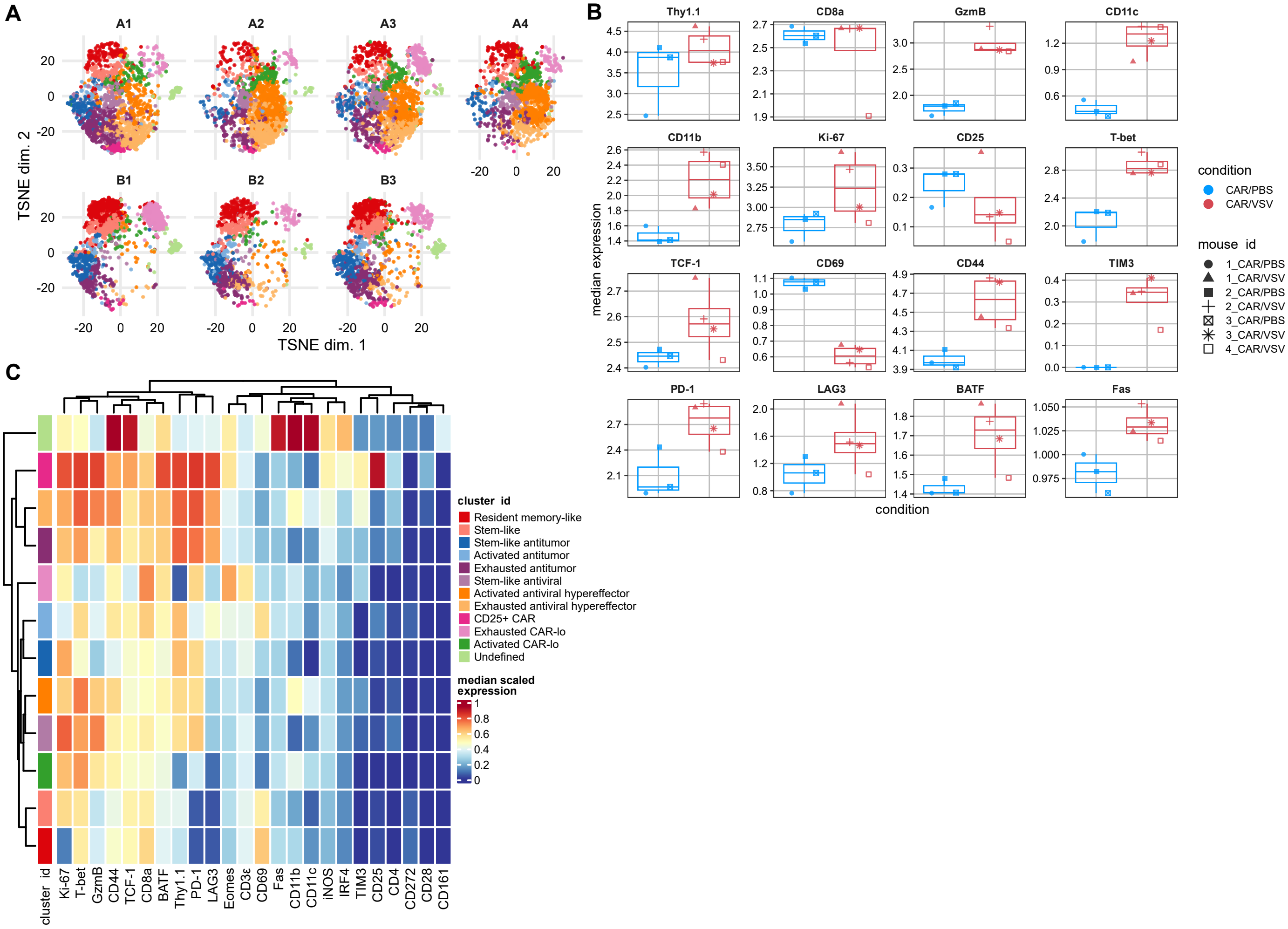

Supplemental Figure 5. Additional data from CyTOF analysis of intratumoral CD8 CAR T cells separated by treatment condition (CAR/PBS versus CAR/VSV). A) Individual TSNE plots separated by sample demonstrating prevalence of each cluster by treatment and sample. B) Quantified expression of markers of interest colored by treatment condition, with point shapes indicating different samples. C) Heat map illustrating expression of markers of interest across the identified clusters.

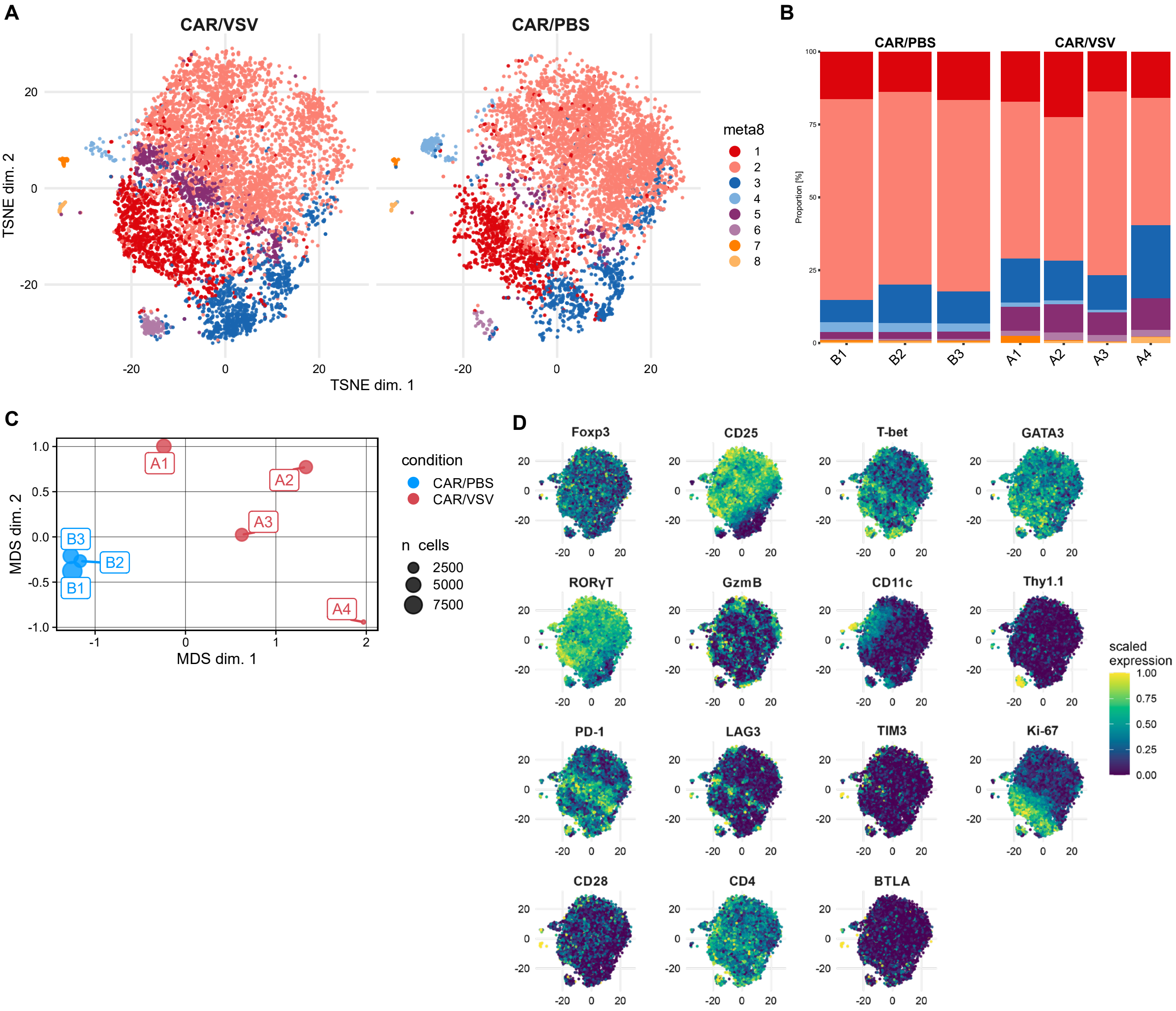

Supplemental Figure 6. VSV cotreatment with CAR T cells also skews the intratumoral CD4 protein profile towards a more engaged, antiviral, Th1-like phenotype. Cytometry by time of flight (CyTOF) was performed on tumor samples from Day 16 of the experiment outlined in Fig. 2A, after enriching for CD45+ cells using magnetic beads. A) Overall TSNE plots of CyTOF data from intratumoral CD4 T cells (endogenous and CAR) for each treatment group combining 4 mice from the CAR/VSV samples and 3 mice from the CAR/PBS samples. B) Relative population abundances for all clusters divided by sample. C) Multidimensional scaling analysis demonstrating population differences between CAR/VSV- and CAR/PBS-treated mice. D) TSNE plots combining all samples across treatment groups colored by expression of individual markers of interest.

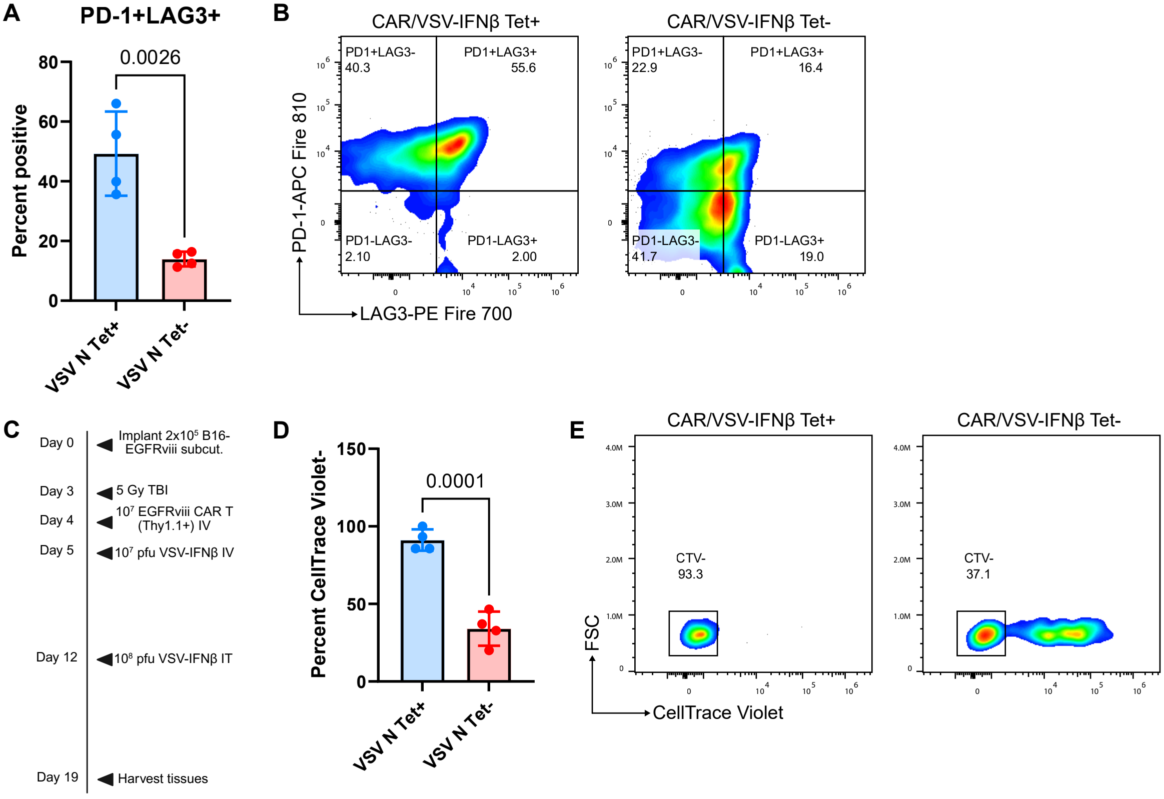

Supplemental Figure 7. TCR-primed CAR T cells express increased levels of PD-1 and LAG3 and undergo enhanced proliferation compared to non-TCR-primed counterparts. A, B) Mice bearing subcutaneous B16EGFRviii tumors were lymphodepleted on day 3 with 5 Gy total body radiation, treated on day 4 with 10^7 EGFRviii CAR T cells, and on day 9 with VSV-mIFNβ intravenously (i.v.) (10^7 pfu). Spleens were harvested on day 16. Quantification and representative flow plots of PD-1 and LAG3 expression between VSV N Tet+ and Tet- CAR T cells (n=4). C) Mice bearing subcutaneous B16EGFRviii tumors were lymphodepleted on day 3 with 5 Gy total body radiation, treated on day 4 with 10^7 EGFRviii CAR T cells stained with CellTrace Violet immediately prior to injection, on day 5 with VSV-mIFNβ intravenously (i.v.) (10^7 pfu), and on day 12 with VSV-mIFNβ intratumorally (i.t.) (10^8 pfu). Spleens were harvested on day 19. D, E) Quantification and representative flow plots of CellTrace Violet negative (highly proliferated) CD8 populations between VSV N Tet+ and Tet- CAR T cells (n=4). Statistical significance was calculated using unpaired two-tailed t-test for 2-sample comparisons.

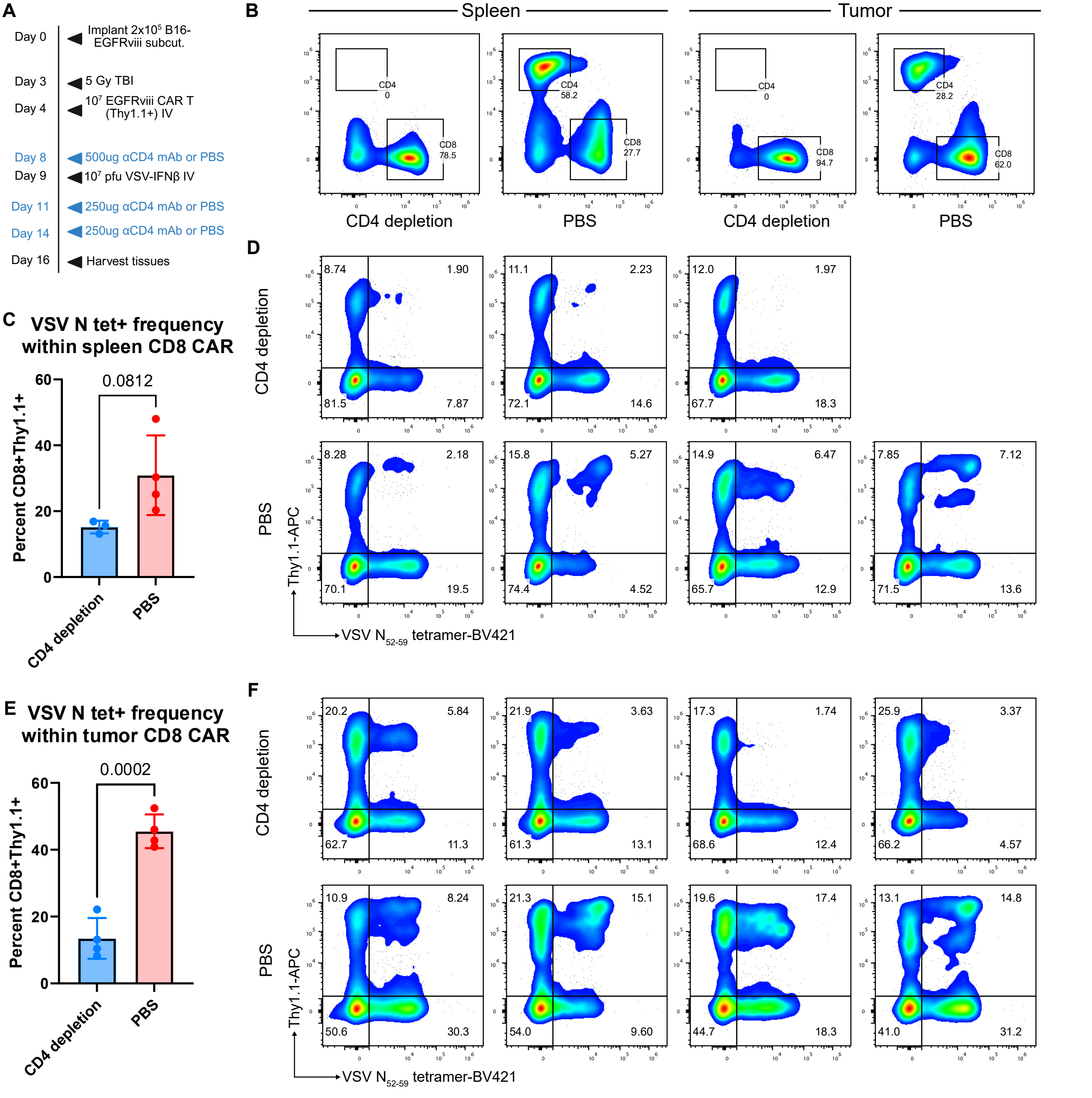

Supplemental Figure 8. Depletion of CD4 T cells during the antiviral immune response diminishes formation of TCR-primed CAR T cells. A) Mice bearing subcutaneous B16EGFRviii tumors were lymphodepleted on day 3 with 5 Gy total body radiation, treated on day 4 with 10^7 EGFRviii CAR T cells, and on day 9 with VSV-mIFNβ intravenously (i.v.) (10^7 pfu). A monoclonal antibody against mouse CD4 or PBS as a control was administered intraperitoneally on days 8, 11, and 14 for selective CD4 depletion. Spleens and tumors were harvested on day 16. B) Representative flow plots of CD3+ T cells from the spleen and tumor confirming CD4 depletion at day 16. C) Quantification of the prevalence of VSV N_{52-59} tetramer+ CAR T cells in the spleen. D) Representative flow plots of CAR (Thy1.1+) and VSV N-reactive (tetramer+) populations in the spleens of mice (n=3-4). E) Quantification of the prevalence of VSV N_{52-59} tetramer+ CAR T cells in the tumor. F) Representative flow plots of CAR (Thy1.1+) and VSV N-reactive (tetramer+) populations in the tumors of mice (n=4). Statistical significance was calculated using unpaired two-tailed t-test for 2-sample comparisons.
